# New insights in polydopamine formation via surface adsorption

**DOI:** 10.1038/s41467-023-36303-8

**Published:** 2023-02-07

**Authors:** Hamoon Hemmatpour, Oreste De Luca, Dominic Crestani, Marc C. A. Stuart, Alessia Lasorsa, Patrick C. A. van der Wel, Katja Loos, Theodosis Giousis, Vahid Haddadi-Asl, Petra Rudolf

**Affiliations:** 1 Zernike Institute for Advanced Materials, University of Groningen, Nijenborgh 4, 9747AG Groningen, The Netherlands; 2 Department of Polymer Engineering and Color Technology, Amirkabir University of Technology, P.O. Box 1587-4413, Tehran, Iran; 3 Electron Microscopy, Groningen Biomolecular Sciences and Biotechnology Institute, University of Groningen, Nijenborgh 7, 9747AG Groningen, The Netherlands; 4 Department of Materials Science & Engineering, University of Ioannina, 45110 Ioannina, Greece; 5 Present Address: Dipartimento di Fisica, Università della Calabria, 87036 Arcavacata di Rende (Cs), Italy

**Keywords:** Surface spectroscopy, Polymerization mechanisms, Bioinspired materials, Molecular self-assembly

## Abstract

Polydopamine is a biomimetic self-adherent polymer, which can be easily deposited on a wide variety of materials. Despite the rapidly increasing interest in polydopamine-based coatings, the polymerization mechanism and the key intermediate species formed during the deposition process are still controversial. Herein, we report a systematic investigation of polydopamine formation on halloysite nanotubes; the negative charge and high surface area of halloysite nanotubes favour the capture of intermediates that are involved in polydopamine formation and decelerate the kinetics of the process, to unravel the various polymerization steps. Data from X-ray photoelectron and solid-state nuclear magnetic resonance spectroscopies demonstrate that in the initial stage of polydopamine deposition, oxidative coupling reaction of the dopaminechrome molecules is the main reaction pathway that leads to formation of polycatecholamine oligomers as an intermediate and the post cyclization of the linear oligomers occurs subsequently. Furthermore, TRIS molecules are incorporated into the initially formed oligomers.

## Abstract (web summary)

Polydopamine is a biomimetic self-adherent polymer, which can be easily deposited on a wide variety of materials but the polymerization mechanism and the key intermediate species formed during the deposition process are still controversial. Here, the authors report a systematic investigation of polydopamine formation on halloysite nanotubes.

## Introduction

Natural phenomena have often served as an inspiration for designing new synthetic materials. In recent years, the extraordinary ability of marine mussels to attach to virtually all types of inorganic and organic surfaces has attracted particular attention^1^. Clues for such universal adhesion ability lie in the high content of catechol (L-3,4-dihydroxyphenylalanine, L-Dopa) as well as primary and secondary amines (lysine and histidine) in the mussel’s adhesive proteins, secreted at the mussel-substrate interface^2^. Inspired by this, Lee et al.^3^ demonstrated that dopamine, an analog of L-Dopa, is prone to undergo self-polymerization to form a thin, surface-adherent polydopamine (PDA) film on a vast variety of materials. The simplest protocol^4^ for coating an object with PDA involves immersing it in an alkaline solution of dopamine and waiting for a PDA layer to form spontaneously on the surface and reach a thickness of typically 1–100 nm. PDA coatings, therefore, overcome the limitations of previous surface modification methods^5^ that require specific substrates or harsh chemical conditions. These unique features of a PDA coating in combination with its high biocompatibility and post-functionalization possibilities have triggered an exponentially growing interest for a broad range of applications^6,7^, including energy storage, environmental remediation, cell encapsulation, and drug delivery.

**Fig. 1:**
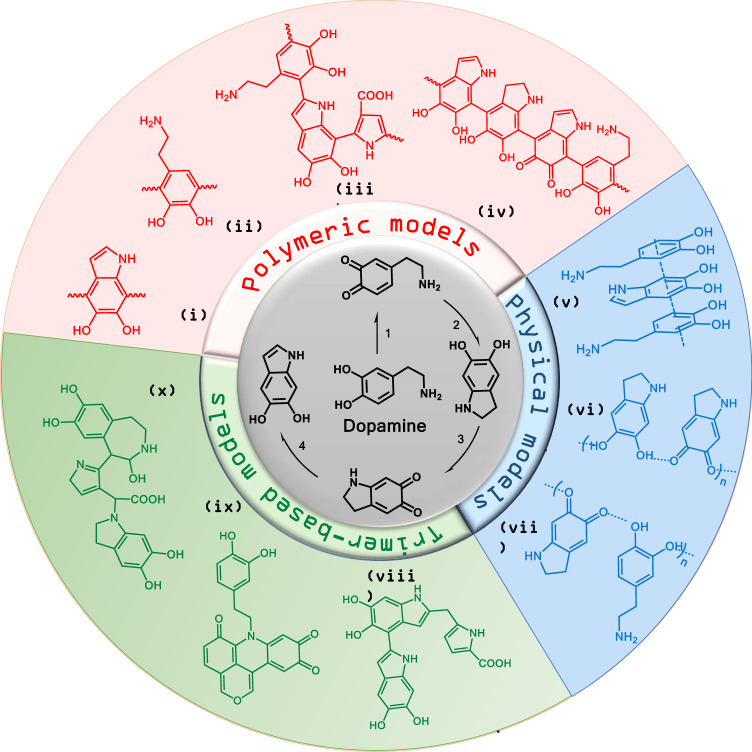
Structural models for polydopamine. In the center: the first step in the polydopamine formation process consists of (1) auto-oxidation of dopamine, (2) intramolecular cyclization, (3) oxidation, and (4) isomerization. For the structures (i)–(x) see text.

Understanding how the PDA coating is formed and what is ultimately its structure, is essential for optimizing its performance for tailored applications. However, its insolubility^8^ in nearly all aqueous and organic solvents complicates the structural evaluation of PDA. In addition, in most cases, the formation of a PDA film on a surface is associated with the formation of PDA particles in solution and it has been shown^9^ that the structure of the film formed on the surface agrees with a process in which particles form in the solution and then adsorb on the surface. These mechanistic complexities as well as the observation that the structural characteristics of PDA films depend on the initial dopamine concentration as well as the type of oxidizing agent used, have led to reports of diverse structures for PDA in literature^10^. The first step of the PDA formation pathway, which comprises oxidation, intermolecular cyclization, and isomerization reactions, has been clearly described by a variety of studies^11^ (central circle in Fig. 1). However, the following step/s leading to PDA formation are still controversial. The proposed structural models for PDA can be divided into three main categories, namely polymeric, physical and trimer-based models, as sketched in Fig. 1. Considering the polymeric models, two primary opposite models were proposed: (a) the “Eumelanin model”, which predicts that PDA formation mimics the synthetic pathway of melanin pigments in living organisms, and where the 5,6-dihydroxyindole (DHI) unit is considered the main building block of PDA (Fig. 1(i))^3^, and (b) the “open-chain poly(catechol/quinone) model” that views PDA as a linear sequence of catecholamine units bonded through biphenyl-type bonds (Fig. 1(ii))^12^. In parallel to these two models, Della Vecchia et al.^13^ have proposed a three-component structure of PDA, which comprises uncycled (catecholamine) and cyclized (indole) units, as well as pyrrole dicarboxylic acid moieties with covalent incorporation of tris(hydroxymethyl)aminomethane (TRIS) (Fig. 1(iii)). Along similar lines, a eumelanin-like polymer chain consisting of DHI units with different degrees of saturation and open-chain dopamine units has also been suggested (Fig. 1(iv))^14^. Moreover, Delparastan et al.^8^ provided evidence for the high-molecular-weight polymeric nature of PDA films wherein the subunits are covalently connected. In contrast to the above studies supporting the existence of covalent bonds between PDA constituents, several researchers have proposed a supramolecular structure for PDA, assembled by physical interactions rather than covalent bonds. For example, using high-performance liquid chromatography (HPLC) and nuclear magnetic resonance (NMR) analysis, Hong et al.^15^ have identified a physical trimer of (dopamine)_2_/DHI formed via self-assembly and stabilized by van der Waals interactions and hydrogen bonds (Fig. 1(v)). Using solid-state spectroscopic techniques, Dreyer et al.^16^ have argued that PDA is a supramolecular aggregate consisting of 5,6-dihydroxyindoline and its quinone derivate, held together by hydrogen-bonding, π-π interaction and charge transfer interactions (Fig. 1(vi)). This view has been supported by Chan^17^, who investigated the PDA formation process by mass spectrometry coupled with isotope-labeling techniques, and provided evidence for non-covalently bound supramolecular aggregates of dopamine and a cyclized intermediate, dopaminechrome (DAC) being the major components of PDA (Fig. 1(vii)). However, most of the studies published in recent years have argued that PDA is mainly composed of oligomers, mainly trimers, formed by covalent coupling oxidation, which bind via non-covalent interactions. Reported observations to support this view include the study performed by Ding et al.^18^, who, based on high-resolution mass-spectroscopy, have proposed a covalently bonded trimer of (DHI)_2_/pyrrole dicarboxylic acid, which links up through non-covalent interactions to build the supramolecular structure of PDA (Fig. 1(viii)). Alfieri et al.^9^ have suggested a polycyclic complex as the main constituent involved in PDA formation rather than DHI-based oligomers (Fig. 1(ix)) and Lyu et al.^19^, also based on a mass-spectroscopy study of PDA formation, have proposed a molecular structure resulting from a complex interplay between dopaminechrome and dopamine units, as the major component of the PDA structure (Fig. 1(x)).

As a consequence of all these discussions, a unified and unambiguous picture of PDA formation has still not emerged in the literature. In addition, most of the previously proposed models for PDA are based on experimental data from mass-spectroscopy methods that are inherently inadequate to discern the chemical structures with similar molecular weights but different functional groups. Accordingly, an investigation of which functional groups are formed and how they change during dopamine polymerization is essential to gain a better understanding of the PDA formation mechanism. Using X-ray photoelectron spectroscopy, Huang et al.^20^ have shown that the functional groups in the PDA film deposited on mica change during the first 5 min of deposition, but remain constant for deposition times >5 min. A similarly fast kinetic behavior was also observed by Zangmeister et al.^21^ and Rella et al.^22^, when they evaluated the surface chemical composition of a PDA film deposited on gold as a function of deposition time.

The aim of this study is to evaluate the kinetics of PDA formation on the surface of a nanometer-sized object because by mixing nano-objects with a large number of adsorption sites into the alkaline solution of dopamine, the adsorption of intermediates species can be enhanced, allowing for better identification. We investigated PDA formation on halloysite nanotubes (HNTs), a low-cost aluminosilicate mineral with a chemical structure of Si_2_Al_2_O_5_(OH)_4_.nH_2_O and a special multi-walled structure where the outer surface is composed of negatively charged tetrahedral SiO_2_, while the octahedral Al_2_O_3_ at the inner surface is positively charged in aqueous solution^23^. The evolution of the PDA layer deposited on the HNTs was monitored by X-ray photoelectron (XPS) and solid-state-nuclear magnetic resonance (ssNMR) spectroscopies, nitrogen adsorption/desorption porosimetry as well as energy dispersive X-ray spectroscopy (EDS). Our results show that the adsorption of the intermediates of dopamine polymerization on HNTs results in a deceleration of the kinetics of PDA formation, which enables the characterization of the intermediates of the reaction. From these data, a mechanism for PDA formation is deduced in which polycatecholamine is an intermediate species.

## Results and discussion

### Time evolution of polydopamine formation on halloysite nanotubes

The exact properties of halloysite nanotubes depend on the specific mineral deposit from which they were mined^23^. A detailed characterization of the pristine HNTs used in this study can be found in Supplementary Fig. 1. To gain insight into the dopamine adsorption on HNTs, an experiment was performed using dynamic light scattering (DLS) measurements where the particle size distribution of HNTs suspension was examined before and after adding dopamine. Since the pH of the solution in this experiment (pH ~5) was not adjusted by adding TRIS no dopamine polymerization was expected. The particle size distributions obtained are shown in Supplementary Fig. 2. HNTs alone show a relatively broad size distribution centered at 320 nm; after adding dopamine to the solution, the particle size distribution shifts to larger values centered at 1200 nm. This increase reflects dopamine adsorption on the HNT surface, as confirmed by the presence of nitrogen from XPS analysis (see Supplementary Fig. 3). Indeed, the negative surface charge of HNTs, their relatively high surface area, and large pore volume favor the adsorption of different compounds ranging from drugs to dye molecules, especially positively charged ones^24^. Considering the pK_a_ values of dopamine (pKa_1_ = 8.9 ± 0.1 and pK_a2_ = 10.5 ± 0.1)^25^ and the pH of the solution (pH ~5), we can deduce that part of the dopamine molecules is protonated and positively charged. This suggests that the electrostatic interactions between protonated dopamine and the negatively charged HNT surface have a leading role in adsorption. Dopamine adsorption results in the compensation of the negative surface charge of the HNTs. Consequently, the nanotubes form larger aggregates because the repulsive interaction between them is reduced.

**Fig. 2:**
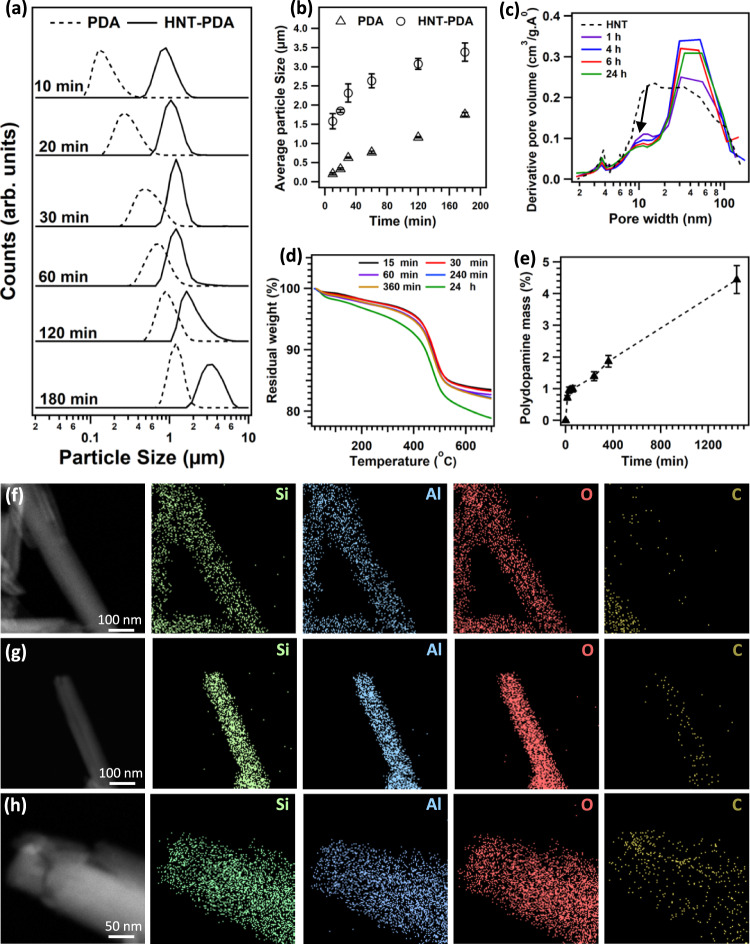
Formation and distribution of polydopamine on HNTs. **a** Particle size distributions—as deduced from dynamic light scattering of a dopamine solution after different polymerization times with and without halloysite nanotubes (HNTs); **b** time evolution of the particle size during polydopamine growth in the presence and absence of HNTs; **c** BJH pore size distribution; **d** TGA graphs for HNTs after different exposure times to the dopamine solution and **e** time evolution of the polydopamine mass deposited on the HNT surface during polymerization. All error bars indicate the standard deviation of the experiments. Scanning transmission electron microscope (STEM) dark-field image and EDS elemental mapping images for HNT after **f** 1 h, **g** 4 h, and **h** 24 h of polydopamine polymerization. The carbon signal in the left bottom corner of **f** is due to the carbon grid that was used as support for the nanotubes. The difference in the intensity of the constituent elements of HNTs is due to the fact that these images were taken on different nanotubes that may have different numbers of layers.

In the next step of our investigation, the impact of the HNTs on the kinetics of polydopamine formation was studied by performing time-dependent DLS measurements. In the first set of experiments, the auto-oxidation of a dopamine solution (10 mM dopamine, 10 mM TRIS at pH 8.5) in the presence of HNTs at a concentration of 0.2 mg/ml was examined. Then the experiment was repeated without adding HNTs in order to establish if the presence of HNTs interferes with the polydopamine particle growth. In both cases a gradual darkening of the solution during the reaction was observed, followed by the precipitation of black insoluble material. However, the darkening proceeded faster in presence of HNTs, suggesting a higher nucleation rate for polydopamine growth. Figure 2a shows the particle size distribution in the dopamine solution after different reaction times for both experiments. At all reaction times, including the initial phase, a monomodal distribution was observed, which gradually shifts towards larger sizes as time increases. The absence of a bimodal distribution implies that the polydopamine particles grow uniformly all the time and do not coexist with oligomers formed in the solution in the initial stages of the reaction. This monomodal distribution is particularly remarkable in the case where HNTs are present because it also indicates that only one type of particle is present. In other words, we do not have a combination of HNTs (coated with dopamine) and separate dopamine-derived particles free in solution. Instead, the data suggest primarily the growth of a polydopamine layer on the nanotubes. The time evolution of the particle size shows that in the absence of HNTs, the dimension of the polydopamine particles is about 180 nm after 10 min of reaction, and gradually increases to 1500 nm after 3 h. When HNTs are present, the particle size is 1500 nm after 10 min of reaction and reaches 3000 nm after 3 h (see Fig. 2b). The small discrepancy observed between Fig. 2a, b is due to the fact that the data shown in Fig. 2b represent the average particle size obtained from triplicated measurements, while data in Fig. 2a is based on a single measurement. These findings suggest that the formation of free-floating oligomers, which has been observed in the initial phase of dopamine polymerization, can be suppressed in the presence of HNTs. From these results, we infer that the oligomers and intermediate species, which are involved in the polydopamine formation, are adsorbed by HNTs. Accordingly, the increase of the average particle size observed during the dopamine polymerization experiment in the presence of HNTs can be the result of nanotube aggregation as a consequence of the intermediate adsorption. Moreover, the adsorption of the intermediates on the HNTs was further confirmed by UV-vis spectroscopy of the reaction mixtures and the results are provided in Supplementary information (see Supplementary Figs. 4, 5).

The impact of dopamine adsorption and polymerization on the porosity of HNTs was evaluated by N_2_ adsorption/desorption analysis for different reaction times; the isotherms are shown in Supplementary Fig. 6, while the Barrett, Joyner, and Halenda (BJH) pore size distributions are depicted in Fig. 2c. The pore size distribution of HNTs changes significantly as the dopamine polymerization proceeds. The feature located at 10–20 nm, attributed to the inner pores of the HNTs, gradually decreases and shifts towards smaller widths (see the arrow in Fig. 2c). This result indicates that the oligomeric and/or polymeric compounds are filling the inner pores of the HNTs as the reaction proceeds. During dopamine polymerization, the pore volume of the macro pores (>50 nm) increases, suggesting that polydopamine deposition on the HNT surface induces the formation of larger aggregates, in agreement with the DLS data discussed above.

To quantify the amounts of organics deposited on the HNTs at different polymerization times, thermogravimetric analysis (TGA) was performed (see Fig. 2d, e). For the derivative graphs, see Supplementary Fig. 7. The gradual decrease in the residual weight after heating up to 700 °C is observed for all samples in Fig. 2d. A non-linear growth of the polydopamine mass deposited on HNTs can be observed in Fig. 2e, pointing to a higher deposition rate in the initial phase of the polymerization, i.e., up to 2 hours of reaction, followed by slower deposition. The steady increase in polydopamine mass during polymerization suggests that longer polymerization times result in larger amounts of polydopamine deposited on the HNT surface, as reported by Mondin et al.^26^ for aluminum oxide particles. Energy dispersive X-ray spectroscopy was employed to investigate the distribution of the deposited organic species on the individual nanotube. Figure 2f–h shows the EDS maps acquired on a single nanotube after 3 different polymerization times, i.e., 1, 4, and 24 h (see Supplementary Table 1 for the atomic percentages). Carbon is detected all over the nanotube in all three samples and the signal becomes more intense as the polymerization time increases, suggesting that the nanotubes are more and more covered by carbon species during dopamine polymerization.

All these observations encouraged us to investigate the chemical structure of the adsorbed species on the HNTs in more detail to better understand the mechanism of polydopamine formation.

### Solid-State ^13^C nuclear magnetic resonance spectroscopy

**Fig. 3:**
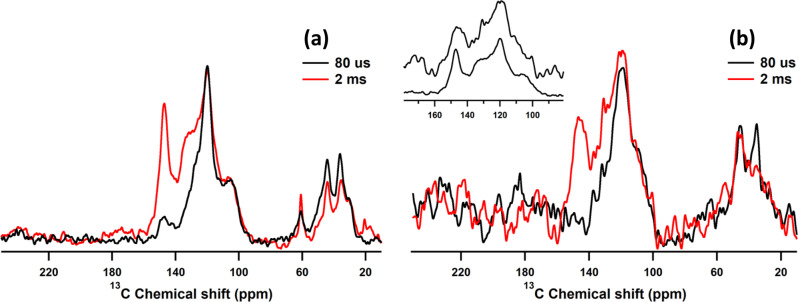
Solid-state NMR of PDA formed in absence and presence of HNTs. **a** Carbon-13 cross-polarization (CP) magic angle spinning NMR spectra for polydopamine formed in the absence of HNTs (PDA-A) after 24 h of polymerization time. Spectra are shown for short (80 μs, black) and long (2 ms; red) CP contact times. The former experiment shows only protonated carbons, where the latter also includes non-protonated carbons. **b** Analogous data for polydopamine deposited on the HNT surface (HNT-PDA), after 24 h. The inset highlights the lack of the peak at 105 ppm in the case of the HNT-PDA sample. These spectra were recorded at 18 kHz MAS on a 600 MHz NMR instrument.

**Fig. 4:**
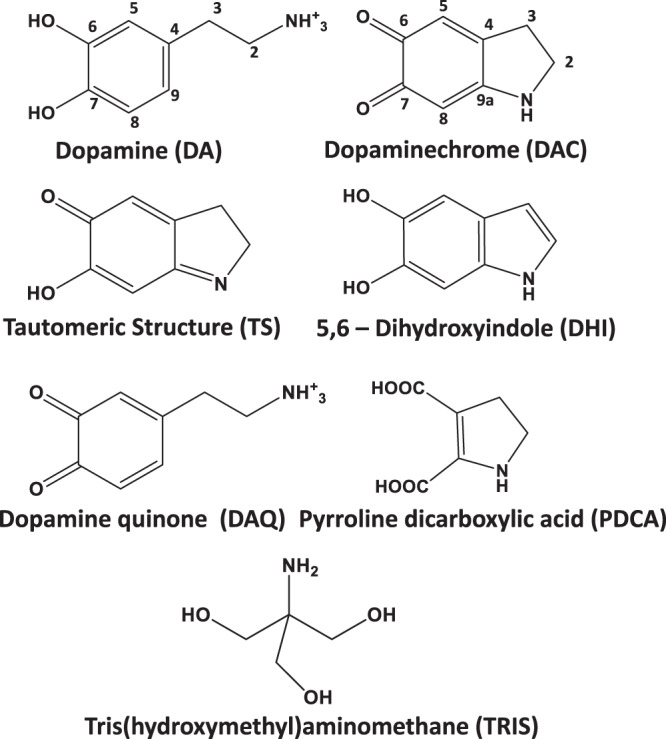
Building blocks of polydopamine. Chemical structures of the expected building blocks of polydopamine. DA and DAQ are shown in their positively charged protonated states, the expected major species in the employed solution conditions.

A first indication that the chemical structure of polydopamine is different when polydopamine aggregates form in solution and when the polymer is deposited on HNTs comes from carbon-13 cross-polarization magic angle spinning nuclear magnetic resonance spectroscopy (CP/MAS ^13^C-NMR). Representative spectra of polydopamine-covered HNTs (HNT-PDA) and polydopamine aggregates (PDA-A) after 24 h of polymerization are shown in Fig. 3. For the analysis of the spectra, we considered the chemical species sketched in Fig. 4 as the expected building blocks for polydopamine, based on the previously accepted proposal that dopamine forms indole/indoline-like structures upon oxidation^11^. In Fig. 3a, the two well-resolved signals around 35 and 44 ppm in the aliphatic region are assigned to the sp^3^ carbons in the uncycled units^27^, i.e. dopamine (DA) and dopamine quinone (DAQ), as well as to carbons at 2 and 3 positions of the indoline ring in dopaminechrome (DAC), its tautomeric structure (TS) and pyrroline dicarboxylic acid (PDCA), see Fig. 4. In addition, a signal at 60 ppm is clearly observed in the spectrum of PDA-A, while the same feature is less pronounced in the spectrum of HNT-PDA (see Fig. 3b). Della Vecchia et al.^13^ observed the same peak and assigned it to the aliphatic carbons of TRIS, which is used to adjust the pH of the reaction. They concluded that polydopamine particles prepared in the presence of TRIS might contain covalently bonded TRIS molecules, especially when the initial dopamine concentration is relatively low.

Small shoulders at ~30 and 48 ppm are identifiable in both spectra of HNT-PDA and PDA-A, confirming that aliphatic carbons become disordered upon dopamine polymerization^27^. This agrees with the aliphatic carbons being present in different chemical structures like DA, DAC, TS, and PDCA in the polydopamine structure. A signal in the range of 170–190 ppm, which is assigned to the resonance of the carbon atoms in carbonyl or carboxylic acid functional groups related to the DAC, TS, DAQ, and PDCA structures^13,14^, would be expected and linked to the oxidative formation of the quinone structures. However, in our ssNMR spectra there is no clear signal at this chemical shift even at the longer CP contact time (2 ms) where non-protonated carbons should be detected. The absence of this peak suggests at best a low amount of this functionality in the sample. Note that this contrasts directly with intermediate species earlier in the reaction process, where clear carbonyl/carboxylic peaks are detected in the 170–190 ppm range (see below).

The signals in the range of 90–160 ppm are assigned to aromatic species. To simplify the interpretation of these resonances, ^13^C CP MAS spectra of the representative samples were acquired with shorter CP contact times, i.e. 80 μs (see Fig. 3, red lines), following the study of Liebscher et al.^14^, which allows the observation of protonated carbons only. As shown in Fig. 3a, four main signals at 105, 119, 130 and 146 ppm are detected in the aromatic region of the polydopamine spectrum acquired with 2 ms contact time, while only two of the four, at about 105 and 119 ppm, are observed with 80 μs contact time. This implies that the latter two signals belong to protonated carbons while the other two belong to quaternary or otherwise non-protonated carbons. The downfield resonance at ~146 ppm can be ascribed to the carbon atoms bonded to oxygen in phenyl form (see the structures of DA, TS, and DHI in Fig. 4). In addition, the bridged carbon atom located at the 9a position in DAC, DHI and TS might contribute to this resonance since it is adjacent to nitrogen and a high downfield shift is commonly observed for such deshielded moieties^14^. Another downfield resonance observed at ~130 ppm is attributed to the quaternary bridged carbon atom in the 4 position of the structures DA, DAQ, DAC, and TS^27^ and to carbon atom in the 6 position of DHI^28,29^. A shoulder in the range of 90–110 ppm can be attributed to aromatic protonated carbons in 3, 5, and 8 positions in DHI^14,27–29^. The observation of such a resonance confirms the presence of DHI in the polydopamine aggregates’ structure, implying that dehydrogenation occurs upon cyclization of the dopamine units. Finally, the signal at 119 ppm can reasonably be assigned to the carbon atoms not discussed so far, i.e. carbons located at 5 and 8 positions of the benzene ring in all the considered structures except DHI. (For more details on chemical shift assignments, see Supplementary Fig. 8).

All the main signals of the polydopamine spectrum are also observed in the spectrum of HNT-PDA (see Fig. 3b). However, the peak (shoulder) representative of DHI in the range of 90–110 ppm is much less pronounced here, suggesting that less DHI is present in the polydopamine deposited on HNTs. The lower contribution of DHI in the HNT-PDA sample compared to the PDA-A sample, was confirmed by subtracting the low CT ^13^C ssNMR spectrum of DHI from the representative spectra, as shown in Supplementary Fig. 9. In addition, the negligible content of DHI during the polydopamine deposition on HNTs was further confirmed by comparing the ^13^C ssNMR spectra of the HNT-PDA at different reaction times with the spectra of starting dopamine and DHI (for more details, see Supplementary Fig. 8). Previous studies reported contradictory findings about the role of DHI in the polydopamine film formation. Ding et al.^18^ investigated the structure of polydopamine deposited on TiO_2_ using TOF-SIMS analysis, finding two main peaks at m/z = 149 and 402; the authors assigned the former to DHI, and the latter to the trimer complex (DHI)_2_/ pyrrole dicarboxylic acid as the main component of polydopamine. However, this proposal is not supported by our findings since (a) two well-resolved signals in the aliphatic region are strong evidence for sp^3^ carbons in HNT-PDA but not present in the structure proposed by Ding et al.^18^; (b) if the (DHI)_2_/pyrrole dicarboxylic acid trimer were the main component of HNT-PDA, we would expect an intense DHI peak in the range of 90–110 ppm in the ssNMR spectrum. This discrepancy can be rationalized considering that mass-spectroscopy cannot distinguish between two chemical structures with the same molecular weight as DAC and DHI, and hence the peak at m/z = 149 in TOF-SIMS spectrum of polydopamine could be also ascribed to DAC. In his study by HPLC coupled with isotope-labeling techniques^17^, Chan provided strong evidence supporting uncycled dopamine and DAC as the main components for polydopamine, in reasonably good agreement with our observations. In addition, Alfieri et al.^9^ found that in contrast to dopamine, DHI cannot form a coating on quartz under the commonly used conditions for polymerization of dopamine (TRIS solution at pH 8.5) and dismissed the decisive role of DHI in the polydopamine film formation. However, the structure containing pyran rings and one tertiary nitrogen proposed by these authors is contradicted by our XPS data of polydopamine deposited on HNTs discussed below, where a noticeable amount of primary amine groups is detected. This raises the question of why the DHI peak is less pronounced in the spectrum of HNT-PDA compared with that of PDA-A. The answer comes from the extremely weak basic strength of DHI molecule (pKa for the strongest base = −6.3)^30^. Such a low basicity ensures that DHI molecules are neutral in the polymerization medium (pH = 8.5), and as a consequence, they cannot associate with the negatively charged HNT surfaces. This can explain the low intensity of the DHI peak for HNT-PDA. Based on this interpretation, the presence of DHI molecules in HNT-PDA has been neglected in our further analysis.

### XPS analysis

In the next step of our investigation, XPS was used to study the PDA film formation kinetics by evaluating the chemical composition of the PDA deposited on the HNT surfaces as a function of the reaction time. In addition, to have a reference, a detailed XPS characterization of the PDA-A sample isolated from an aqueous alkaline dopamine solution after 24 h can be found in the Supplementary Fig. 10. In parallel, to investigate the effects of the substrate dimensions on the kinetics of PDA film formation, a substrate with macroscopic dimensions (1.5 × 1.5 cm^2^) and a chemical nature similar to the external surface of HNTs, namely a Si wafer covered by an oxide layer, was exposed to the dopamine polymerization medium for different times, and the obtained PDA films, denoted with SiO_2_-PDA in the following, were also analyzed by XPS. The survey spectra of HNT-PDA and SiO_2_-PDA as a function of deposition time are shown in Supplementary Fig. 11. All samples exhibit carbon and nitrogen signatures, indicating the formation of a polydopamine layer on the surface. The stoichiometric analysis was performed by collecting the detailed core level spectra of the constituent elements of HNTs, SiO_2_, and polydopamine, i.e. Al2*p*, Si2*p*, O1*s*, C1*s,* and N1*s*, and deducing the corresponding atomic percentages in the probed volume in order to evaluate the evolution of the elemental composition as a function of dopamine polymerization time (Supplementary Table 2). For all samples, the atomic percentage of Al and/or Si decreases continuously with increasing deposition time, while the atomic percentages of nitrogen and carbon rise, indicating PDA film growth on the surface. In addition, the N/C atomic ratio (0.10 ± 0.02) in all samples is in agreement with the value of polydopamine (N/C = 0.10), confirming the presence of PDA on the surface.

**Fig. 5:**
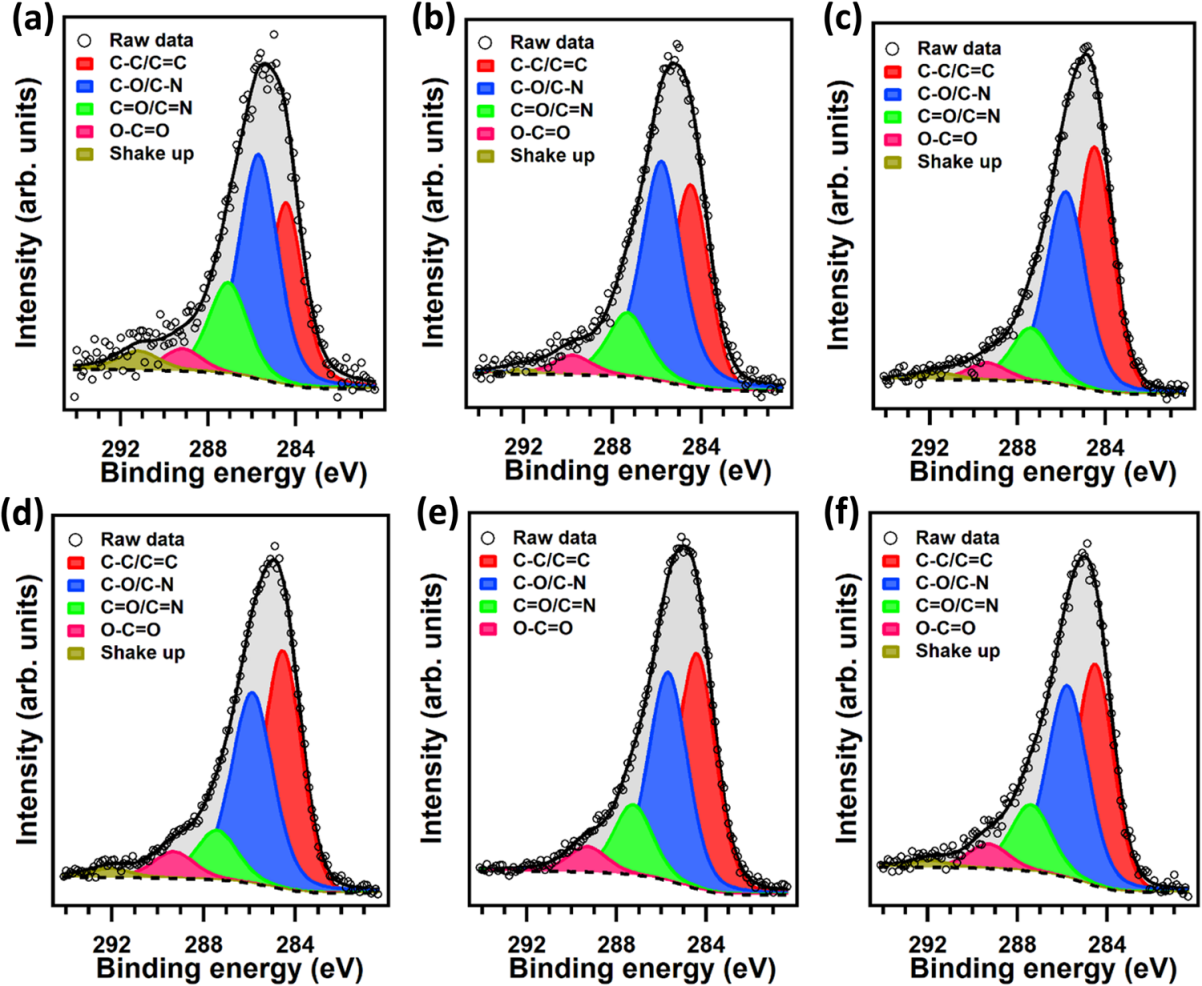
XPS characterization of modified HNT-PDA samples. XPS spectra of the C1*s* core level region for HNT-PDA after **a** 5 min, **b** 15 min, **c** 30 min, **d** 60 min, **e** 120 min, and **f** 240 min of polydopamine polymerization.

Typical detailed XPS spectra of the C1*s* core level region for HNT-PDA samples are shown in Fig. 5. The same chemical species as observed in the C1*s* spectrum of PDA-A (see Supplementary Fig. 10b) can be identified in the C1*s* spectra of HNT-PDA samples, namely C-C/C = C (red, observed at binding energy (B.E.) of 284.8 eV), C-O/C-N (blue, B.E. = 286.1 eV), C = O/C = N (green, B.E. = 287.5 eV), O = C–O (pink, B.E. = 289.2 eV) bonds and shake-up component at a BE of 291.3 eV^18,31,32^.

**Fig. 6:**
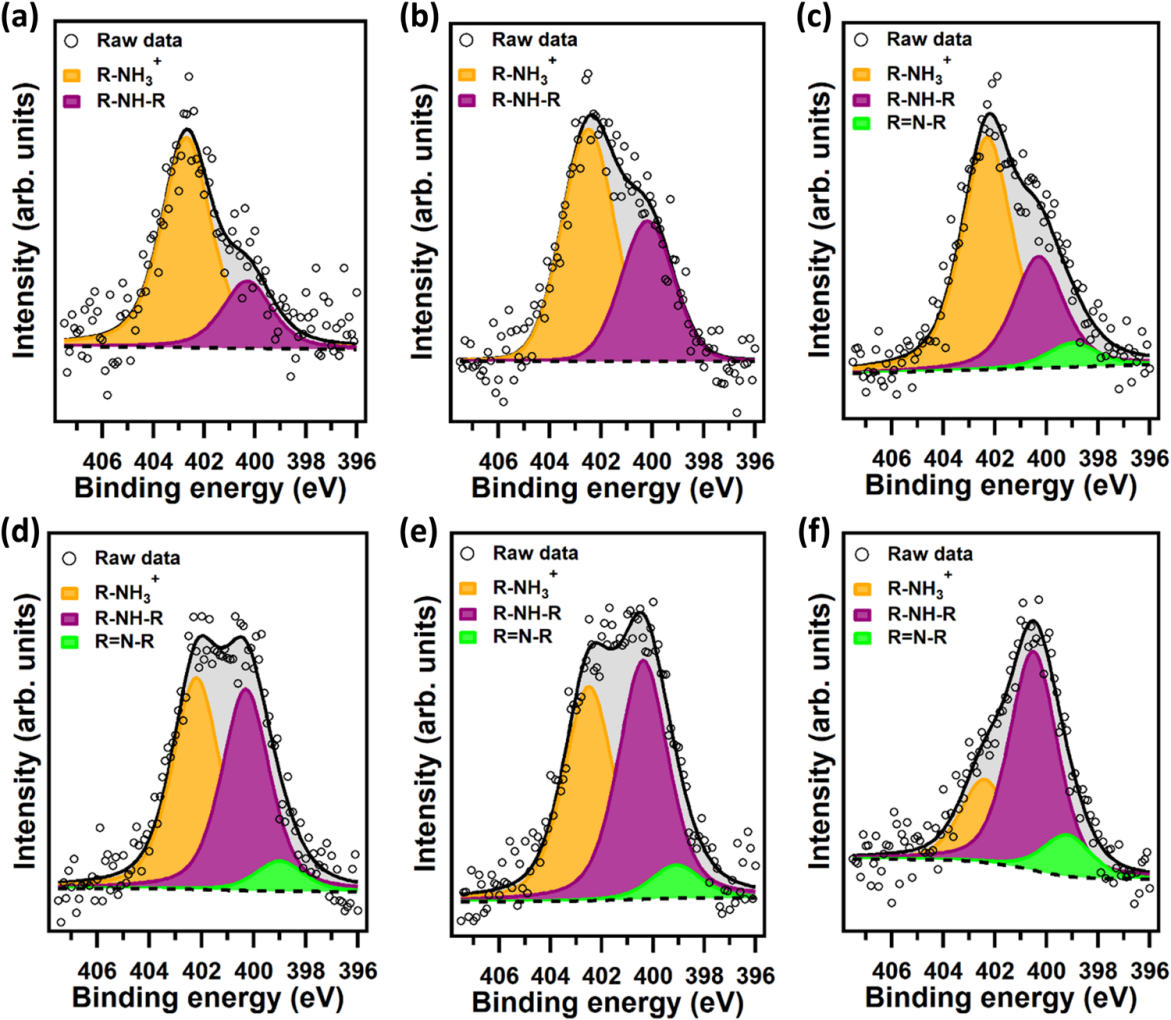
XPS analysis of modified HNT-PDA samples. XPS spectra of the N1*s* core level region for HNT-PDA after **a** 5 min, **b** 15 min, **c** 30 min, **d** 60 min, **e** 120 min, and **f** 240 min of polydopamine polymerization.

The spectrum of the N1*s* core level region changes significantly as the polymerization proceeds (see Fig. 6). In the initial phase, the N1*s* peak can be fitted with two components, attributed to R-NH_3_^+^ (marked in orange in Fig. 6, B.E. = 402.5 eV) and R-NH-R (purple, B.E. = 400.1 eV) moieties^18,33^. Considering the building blocks shown in Fig. 4, a primary amine component points out the presence of both DA and DAQ, while secondary amines feature in DAC and PDCA. However, as the reaction proceeds, a third component appears at B.E. = 398.8 eV, which is the characteristic B.E. of an imine structure (green)^31^.

**Fig. 7:**
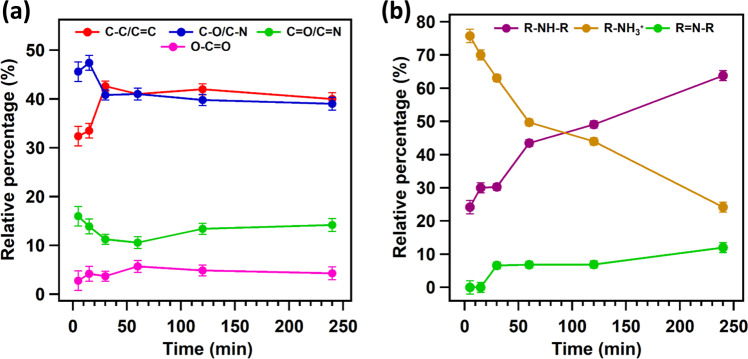
Evolution of carbon and nitrogen species during polymerization. Time-dependent evolution of **a** C1*s* and **b** N1*s* components for HNT-PDA during dopamine polymerization. The error bars represent the standard deviation of the experiments.

Figure 7a, b shows the evolution of the relative percentages of carbon and nitrogen species during the polymerization as deduced from their photoemission intensities. It is worth noting that the ratio between C-C/C = C and C-O/C-N components is below 1 at the beginning of the reaction, i.e. after 5 min and 15 min of reaction time, while it stabilizes at 1 as the polymerization proceeds (see Fig. 7a). This finding is somewhat surprising; indeed, considering only the structures originating from dopamine, i.e. DAQ, DAC, TS and PDCA, this ratio should not be less than 1. Its lower value is therefore a robust indicator for the presence of another molecular structure with alcohol or amine functional groups in the initial phases of the polymerization, namely the TRIS molecule. This hypothesis was confirmed by performing a similar experiment using a carbonate buffer instead of a TRIS buffer; for more details see Supplementary Fig. 12. Della Vecchia et al. reported similar observations^13,34^, identifying incorporation of TRIS into the polydopamine structure obtained from an aged alkaline solution of dopamine after 24 h of reaction. The most striking result that emerges from the curve fitting of the N1*s* spectra is that in the initial phase of polymerization, primary amine is the main component, while the secondary amine contribution becomes more pronounced with increasing reaction time (see Fig. 7b).

The prominent primary amine component confirms the presence of intact TRIS units at the beginning of the reaction, which corroborates the interpretation of the C1*s* spectra. In addition, as the secondary amine component becomes more important, the imine feature starts to be noticeable in the N1*s* spectra and its intensity continuously increases as the polymerization proceeds. These findings led us to conclude that the imine component is attributed to TS, which is a tautomer of DAC. It should be noted that the spectra of the C1*s* and N1*s* core level regions for HNT-PDA samples after 240 min of reaction are identical to those of PDA-A samples, suggesting that the surface charge of HNTs does not affect the distribution of functional groups in the PDA film deposited on the surface. Based on the above results, we can infer that the main building blocks of the polydopamine film are DA, DAQ, DAC, PDCA, TS, and TRIS. Surprisingly, in contrast to HNTs, after deconvolution of the N1*s* and C1*s* spectra for SiO_2_-PDA samples, no significant changes in the relative amounts of nitrogen and carbon species were observed during the reaction; all samples show relative percentages comparable to PDA aggregates (Supplementary Fig. 13 and Table 3). As already mentioned in the introduction, Huang et al.^20^ systematically monitored the growth of a PDA film on a mica surface and observed an evolution of the N1*s* and C1*s* spectra as a function of reaction time close to ours for HNT-PDA; however, in their case, 60–300 s were sufficient for the formation of a complete PDA layer with a thickness ranging between 0.5 and 1.1 nm. In the case of our oxidized Si substrate, we do not observe this evolution. We suggest that this is due to the much lower number of adsorption sites on the small SiO_2_ surface (2.25 × 10^−4^ m^2^) as compared to the much larger surface (18.6 m^2^ for 0.4 g HNT) present in the solution when HNTs are added: very few of the intermediates and oligomers associated with the PDA aggregation can be adsorbed on the oxidized Si surface, while the majority of them form PDA aggregates in solution, which consequently deposit on the substrate. However, when HNTs are present, the amount of the available adsorption sites are so large that intermediates and oligomers adsorb on the nanotubes to such an extent that we can no longer detect PDA aggregates in solution. On the HNT surface, we can therefore follow the PDA polymerization and unravel the mechanism.

### Towards a rational model for the formation of polydopamine

To determine which intermediates are formed in PDA polymerization, and therefore understand how the polymerization proceeds, an empirical model was developed based on the analysis of the XPS data. The relative percentage of each building block was calculated by using a numerical method. Further details on the model development and the calculations are presented in the Supplementary information.

**Fig. 8:**
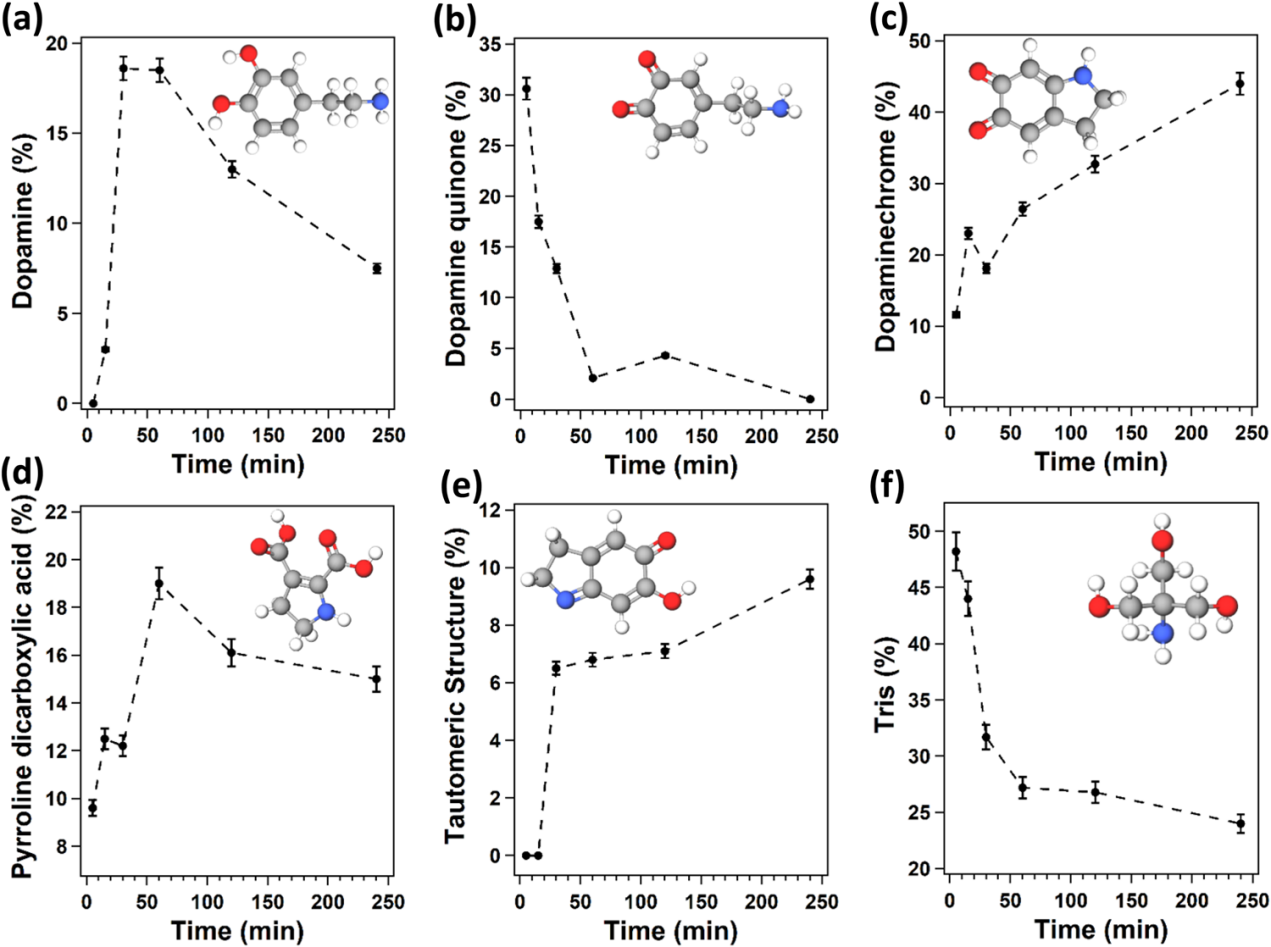
Relative percentage of polydopamine building block during film formation. Time evolution of the concentration of the main building blocks as sketched in the inset during polydopamine film formation on halloysite nanotubes as deduced from the numerical model for: **a** dopamine, **b** dopamine quinone, **c** dopaminechrome, **d** pyrroline carboxylic acid, **e** tautomeric structure and **f** Tris. The error bars represent the standard deviation of the experiments. For details, see the text and Supplementary information.

Figure 8 shows the relative percentage of each building block during the formation of the polydopamine film. In Fig. 8b, one sees a high amount of dopamine quinone present after 5 min of polymerization; this is expected according to the commonly accepted mechanism for polydopamine formation, where the first step is the auto-oxidation of dopamine (DA) to dopamine quinone (DAQ) in an alkaline solution^11^. However, as the reaction proceeds, the amount of DAQ decreases, suggesting that this molecule is consumed during polymerization.

In addition, as illustrated in Fig. 8a, the amount of dopamine (DA) in the deposited film increases in the initial phases of the reaction, but starts to decrease after 60 min of polymerization. This mirrors the characteristic result of kinetics of intermediate species in consecutive reactions, where a certain molecule formed in the first reaction is consumed in the second one. Accordingly, we propose that the reaction responsible for increasing the dopamine concentration in the film structure is the crosslinking of quinone (DAQ) and DA molecules through a reverse dismutation reaction resulting in the formation of polycatecoholamine^35^. For more details on the mechanism of this reaction see Supplementary Fig. 14. The second reaction consists then of the oxidation and cyclization of polycatecoholamine leading to the creation of dopaminechrome (DAC) units, as confirmed by the increase of that building block seen in Fig. 8c. The gradually decrease of DAQ and the increase of DAC structures in the oligomers adsorbed on HNTs were further verified by UV-vis reflection spectroscopy on the nanotubes in the solid-state (For more details see Supplementary Fig. 15). Still, Fig. 8d shows an increase as a function of polymerization time also for pyrroline dicarboxylic acid (PDCA), suggesting that PDCA also originates from DAC. This is consistent with the study of Napolitano et al.^36^, which revealed the possibility of oxidative breakage of o-quinone rings resulting in the formation of pyrrole dicarboxylic acid species. However, the rate at which PDCA increases is less than that of DAC, indicating that only a small portion of dopaminechrome units undergoes oxidative ring breakage. In addition, if one considers Fig. 8e, one sees that the increase in TS during polymerization identifies this building block as a product of DAC tautomerization. Finally, Fig. 8f shows that TRIS is more prominently present at the beginning of the reaction, suggesting the adsorption of the TRIS units by hydrogen bonds and/or electrostatic interaction on the HNT surface. The growth of the polydopamine layer on the HNTs leads to a decrease of TRIS, as the reaction proceeds.

**Fig. 9:**
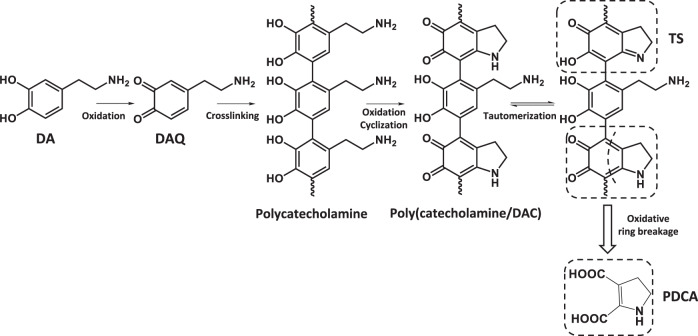
Polydopamine film formation. Proposed model for polydopamine film formation.

Overall, all these results allow us to draw a complete picture of the formation of the polydopamine film as illustrated in Fig. 9. As a first step, dopamine molecules (DA) undergo a pH-induced auto-oxidation reaction resulting in quinone molecules (DAQ) formation. Then crosslinking of DAQ molecules occurs through the formation of biphenyl bond, which leads to polycatecholamine as an intermediate for the film growth process. These results corroborate the findings of Burzio et al.^37^ who reported that the *o*-quinone groups can react through reverse dismutation with catechol groups to produce two semiquinone radicals that couple to form di-catechol crosslinks. Then uncycled units in the polycatecholamine undergo simultaneously oxidation and intermolecular cyclization to generate dopaminechrome. In the next step, the tautomerization of DAC molecules causes the appearance of a small quantity of TS molecules in the polydopamine film. Lastly, the oxidative ring breakage reaction of DAC generates PDCA units, which can be detected in low amounts in the polydopamine film. It is noteworthy that the model proposed in this study implies an important role of the quinone and dopachrome species in dopamine polymerization, which agrees well with the steps involved in the biosynthesis pathways of the melanin pigments^38^.

In conclusion, this study presents a systematic investigation of polydopamine film formation on the nanometer-sized surface of halloysite nanotubes. The role of 5,6-dihydroxyindole molecules in PDA film formation process was ruled out by a comparative inspection of the ssNMR spectra of PDA film formed on the HNTs and that of PDA aggregates. Interestingly, monitoring the process kinetics showed that the presence of HNTs in the dopamine polymerization medium resulted in a deceleration of the PDA film formation process because of the massive adsorption of intermediates onto HNT surfaces. As a consequence of such a decelerated process, the intermediates involved in the formation of the PDA film could be identified and a complete picture of the mechanism of the process could be drawn up. Our results show that oxidative coupling of the quinone units leading to the formation of the polycatecholamine species is the most dominant reaction in the initial phase of the film formation process. As the reaction time proceeds, intermolecular cyclization gradually occurs in the dopamine units that are present in the polycatecholamine species. In addition, we could provide experimental evidence for the inclusion of TRIS in the layer during the initial phase of the film formation process, while the presence of this molecule becomes less important as the reaction time proceeds. The results presented here give new insights into the PDA film formation, which can be further exploited for tuning the properties and functions of the PDA coating.

## Methods

### Materials

Halloysite nanotubes (HNT, Al_2_Si_2_O_5_(OH)_4_.2H_2_O), dopamine hydrochloride (DA, C_8_H_11_O_2_N·HCl, ≥98%, AR), tris(hydroxymethyl)aminoethane (TRIS, C_4_H_11_O_3_N, ≥99.9%, AR) were purchesed from Sigma Aldrich and 5,6-diyroxyindole (DHI, C_8_H_7_NO_2_, product number CDS021567) was purcehsed from Sanbio. All the chemicals were used as received and all the solutions were prepared using Milli-Q water.

### Preparation of PDA aggregates

PDA aggregates were synthesized by adding 0.4 g of DA to 200 ml of 10 mM TRIS solution at pH = 8.5. The color of the solution gradually changed from colorless to black, indicating the formation of PDA. After 24 h of stirring at ambient temperature, the black precipitate was separated by centrifugation and dried in a vacuum jar. This product is labeled as PDA-A.

### PDA coating procedure

0.8 g of HNTs were added to 200 ml of 10 mM TRIS aqueous solution at pH = 8.5. The mixture was sonicated for 30 min to assure good dispersion, then 0.4 g of DA was added to the suspension, followed by stirring at room temperature for different time intervals, namely 5, 15, 30, 60, 120, and 240 min. The product was separated by centrifugation and washed several times with Milli-Q water to remove the unreacted reagents. Then, the obtained pellet was dried under vacuum overnight; it is denoted as HNT-PDA. To study the effects of the TRIS buffer on the initally adsorbed oligomers on HNTs, the same procedure was performed using a carbonate buffer aqueous solution (10 mM, pH = 8.5) instead of the TRIS buffer solution. The samples were withdrawn after 5 and 15 min of the reaction, centrifuged, washed several times with Milli-Q water, and dried under vacuum overnight. In a complementary experiment, in order to investigate the effects of substrate dimensions on the PDA formation process, Si wafers covered by a native oxide layer (Siegert Wafer, thickness 525 ± 20 μm) with dimensions of 1.5 × 1.5 cm^2^ were immersed in a 50 ml of a freshly prepared solution of DA (2 mg/ml) and TRIS (10 mM). The reaction mixture was then shaken at 150 rpm and ambient temperature using an orbital shaker incubator (VWR, Incubating Mini Shaker). After certain time intervals, the substrate was removed from the solution, rinsed several times with Milli-Q water, and gently dried with an argon flow. The coated substrate is referred to as SiO_2_-PDA.

### Characterization

Dynamic light scattering (DLS) measurements were conducted using a Malvern Zetasizer Ultra. Stock solutions of DA (20 mg/ml) and TRIS (1 M) were prepared and filtered through a 0.2 μm pore size filter to remove the non-dissolved species. 0.1 ml of the DA stock solution was diluted to 1 ml with Milli-Q water or an HNT suspension at a concentration of 0.2 mg/ml. Then, 10 μl of the TRIS stock solution was added to start the dopamine polymerization. After a certain time of stirring, the reaction was stopped by acidifying the solution to pH = 2 using a 4 M HCl solution. Acidified solutions were then transferred to the dust-free disposable polystyrene cuvettes for DLS measurements. It is noteworthy that for non-spherical particles, the particle size obtained from DLS analysis represents the size of spherical aggregates^32^. The UV-vis spectroscopy of the reaction supernatant samples was performed using a HITACHI Spectrophotometer (U-1800). The samples were withdrawn from the reaction mixture after different times of reaction, filtered by 0.2 μm pore size filter, and diluted to 0.2 mg/ml with Mili-Q water before measuments. The diffuse reflectance spectra of HNTs samples on barium sulfate coatings were aquired using a Shimadzu UV-VIS-NIR Spectrophotometer (UV-3600) equipped with an integrating sphere attachment. The mass percentage of the PDA layer deposited on the HNTs was studied through TGA by heating samples of approximately 5 mg at a rate of 5 °C/min from 25 to 700 °C under N_2_ atmosphere, using a PL TGA (Polymer laboratories, TGA 1000, UK). Porosimetry studies were carried out using a Micrometrics ASAP 2420 V2.05 instrument and the N_2_ adsorption/desorption isotherms were acquired at −196 °C. The Barrett, Joyner, and Halenda (BJH) model was applied to the N_2_ desorption data to obtain the pore size distribution. A Tecnai T20 transmission electron microscope (TEM), operating at 200 keV, was utilized to investigate the structure of the nanotubes. For this HNT powder was sonicated in ethanol and the suspension dropcast on a holey carbon-coated gold grid. Energy dispersive X-ray spectroscopy (EDS) was performed using an Oxford instruments Xmax T80 silicon drift detector on the same electron microsope using the STEM mode with high angle anular dark-field imaging. Solid-state-nuclear magnetic resonance (ssNMR) was performed on a Bruker AVANCE NEO 600 MHz (14.1 T) spectrometer, using a 3.2-mm EFree HCN MAS probe from Bruker Biospin, at 18 kHz MAS and 283 K temperature. 13 C ssNMR spectra were acquired using 13C–1H 70–100 ramped MAS cross-polarization (CP) ssNMR experiments, with 50 kHz nutation frequency for 13 C and around 80 kHz for 1H. Two distinct experimental conditions were used for the two investigated samples: short and long CP contact time, corresponding to 80 μs and 2 ms. The free induction decays were recorded under high-power proton decoupling (100 kHz) using the two-pulse phase modulation scheme (TPPM)^39^, with a recycle delay of 2.5 s and by averaging 1k and 20k transients for PDA-A and HNT-PDA respectively. The ssNMR samples at different time points were prepared analogous to the preparation conditions described for HNT-PDA, and corresponding ssNMR experiments were recorded by averaging 16k transients. NMR spectra were processed with NMRPipe^40^. Chemical shifts were referenced to 4,4-dimethyl-4-silapentane-1-1 sulfonic acid using the adamantane ^13^C chemical shifts as an external reference, as previously described^41,42^. A Surface Science SSX-100 ESCA instrument with a monochromatic Al Kα X-ray source (hv = 1486.6 eV) operating in a vacuum of 2 × 10^−9^ mbar was used to perform the X-ray photoelectron spectroscopy (XPS) analysis. Samples were prepared ex situ by pressing dried powders of PDA-A or HNT-PDA onto silver substrates (previously prepared by flattening Ag pearls (Goodfellow, silver lump AG006100, purity 99.999%, size: 3 mm) in a press (RHC, 30-ton pillar press)). Data acquisition was performed on a spot diameter of 1000 μm and the electron take-off angle with respect to surface normal was 37°. The energy resolution was set to 1.26 eV for both the survey spectra and the detailed spectra of the C1*s* and N1*s* core level regions; a charge neutralizer system in optimized conditions was used during the XPS measurements to compensate for charging effects. Binding energies are reported ±0.1 eV and referenced to the Si2*p* photoemission peak centered at a binding energy of 102.7eV^43^. The detailed spectra were analyzed with the help of the least-squares curve-fitting program Winspec (University of Namur, Belgium). Deconvolution of the spectra included a Shirley baseline subtraction and fitting with peak profiles taken as a convolution of Gaussian and Lorentzian functions. The uncertainty in the peak intensity determination is within 2% for all core levels reported. All measurements were carried out on freshly prepared samples and on three spots in order to check for homogeneity.

## Supplementary information

Supplementary Information

## Data Availability

The authors declare that all the relevant data supporting the findings of this study are available within the article and its Supplementary information files. All raw data generated during the current study are available from the corresponding authors upon request.
