# Supplementary material for: New insights in polydopamine formation via surface adsorption
Source: Nat Commun. 2023 Feb 7;14:664. doi: 10.1038/s41467-023-36303-8 (PMC9905603; doi:10.1038/s41467-023-36303-8)
Supplement: Supplementary file 1 — Supplementary Information [file 41467_2023_36303_MOESM1_ESM.pdf]

Supplementary Information

New insights in the polydopamine formation via surface adsorption

Hamoon Hemmatpour[1,2], Oreste De Luca[1✉,5], Dominic Crestani[1], Marc C.A. Stuart[3,] Alessia Lasorsa[1], Patrick C.A. van der Wel[1], Katja Loos[1], Theodosis Giousis[1,4], Vahid Haddadi-Asl[2] and Petra Rudolf[1✉]

1 Zernike Institute for Advanced Materials, University of Groningen, Nijenborgh 4, 9747AG Groningen, the Netherlands

2 Department of Polymer Engineering and colour Technology, Amirkabir University of Technology, P.O. Box 1587-4413, Tehran, Iran

3 Electron Microscopy, Groningen Biomolecular Sciences and Biotechnology Institute, University of Groningen, Nijenborgh 7, 9747AG Groningen, The Netherlands

4 Department of Materials Science & Engineering, University of Ioannina, 45110, Ioannina, Greece.

✉email: oreste.deluca89@gmail.com, p.rudolf@rug.nl

5 Present address: Dipartimento di Fisica, Università della Calabria, 87036 Arcavacata di Rende (Cs), Italy

Supplementary Figures

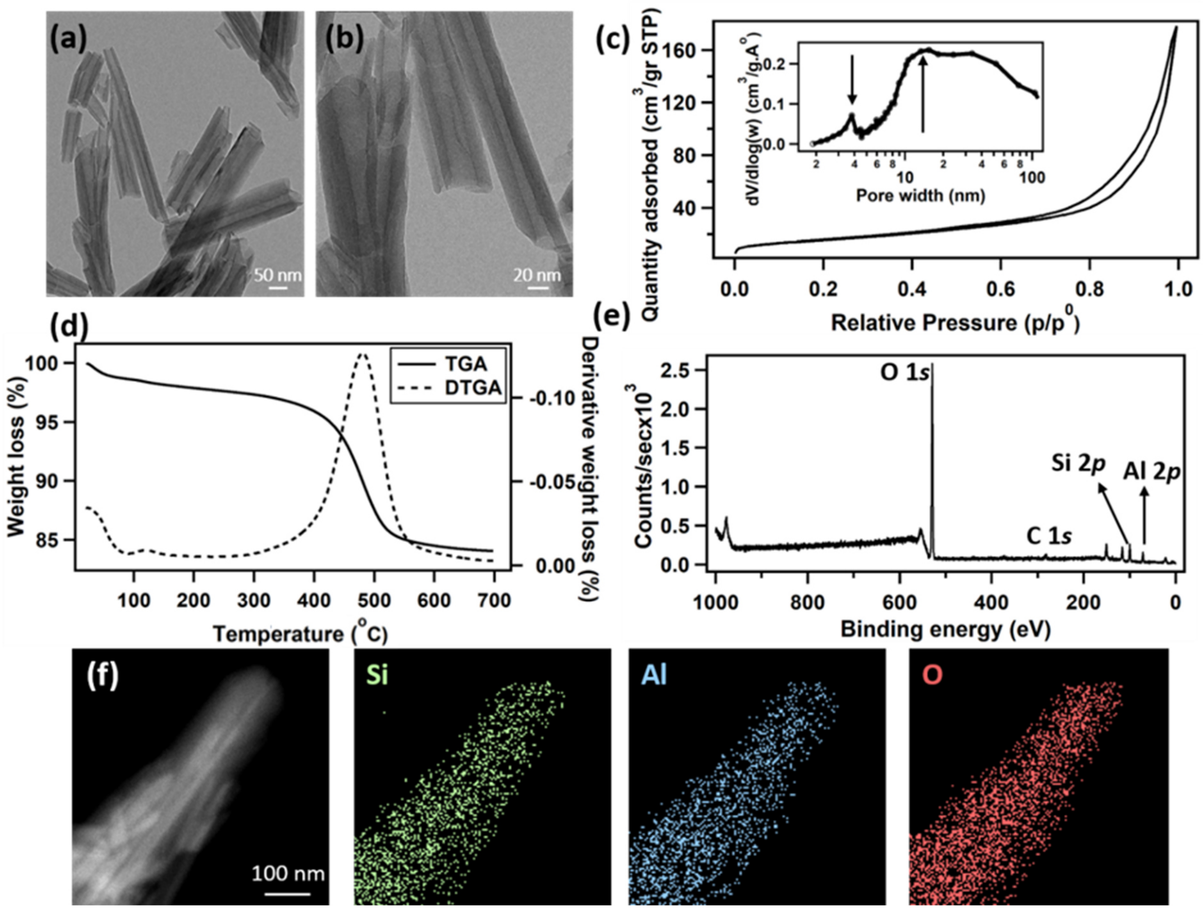

Supplementary Figure 1. Characterization of pristine halloysite nanotubes. (a and b) Transmission electron microscopy images; (c) N_2 adsorption/desorption isotherm; inset: pore size distribution, (d) thermogravimentric and differential thermogravimetric analysis; (e) X-ray photoelectron spectroscopy wide scan spectra and (f) STEM dark-field image and EDS elemental mapping images from energy dispersive X-ray spectroscopy.

As seen in the transmission electron microscopy (TEM) images in Supplementary Fig. 1a-b, the HNTs used in this study have an open-ended tubular structure with an inner diameter of 5 - 20 nm, an outer diameter range from 50 to 100 nm and a length in the range of 0.5 – 1.5 µm. To learn about their porosity, N_2 adsorption and desorption was analysed, and as shown in Supplementary Fig. 1c, the isotherm of HNT can be categorized as type IV according to the IUPAC classification, which points to a mesoporous structure.[1] BET analysis showed that the HNT surface area and total pore volume are 46.6 m^2/g and 0.28 cm^3/g, respectively. Such relatively high surface area and large pore volume make HNT an ideal candidate as sorbent of a vast variety of molecules.[2] When examining the logarithmic pore size distribution deduced from the desorption branch of the N_2 isotherm based on the Barrett, Joyner, and Halenda model (see inset in Supplementary Fig. 1c), two main pore size distributions can be distinguished; the first peak centred at 3.5 nm is assigned to the longitudinal slit-shaped pores on the outer surface of HNTs. According to Koyama and co-workers[3], such mesopores form

during the dehydration process when the rolled-up layers of the tubular halloysite separate. The second wide feature in the pore size distribution between 6 and 100 nm comprises the signature of two different structural characteristics; the shoulder centred at 15 nm is assigned to the cavity inside the nanotubes, while the macropores in the range of > 50 nm relate to the randomly arranged empty spaces outside the nanotubes, formed when they agglomerate into bundles. When the thermal decomposition of HNTs was studied by thermogravimetric analysis (TGA), we found a two-step thermal decomposition behaviour (see Supplementary Fig. 1d); in the first step, occurring at temperatures between 100 and 150 °C, the residual water intercalated in the HNT interlayer space is removed, resulting in a small weight loss of about 1 wt.%. The second step from 400 to 600 °C is due to the dihydroxylation of Al-OH groups on the HNTs' inner surfaces[4] and results in a weight loss of about 13.6 wt.%. The chemical composition of HNT was investigated by X-ray photoelectron spectroscopy (XPS) and elemental mapping through energy dispersive X-ray spectroscopy (EDS) and the results are presented in Supplementary Figures 1e and 1f respectively. The XPS survey scan shows the signatures of the elements expected for aluminosilicate, i.e. O, Si and Al; in addition, a small feature typical of adventitious carbon is observed.[4] Stoichiometric analysis of the detailed core level XPS data reveals that the Si/Al atomic ratio is 1.1±0.1, in good agreement, within the experimental uncertainty, with the chemical formula of HNT. The homogenous distribution of Si, Al and O over an individual HNT was confirmed by EDS elemental mapping (see Supplementary Fig. 1f). Furthermore, the carbon signal was not detected on the individual nanotube.

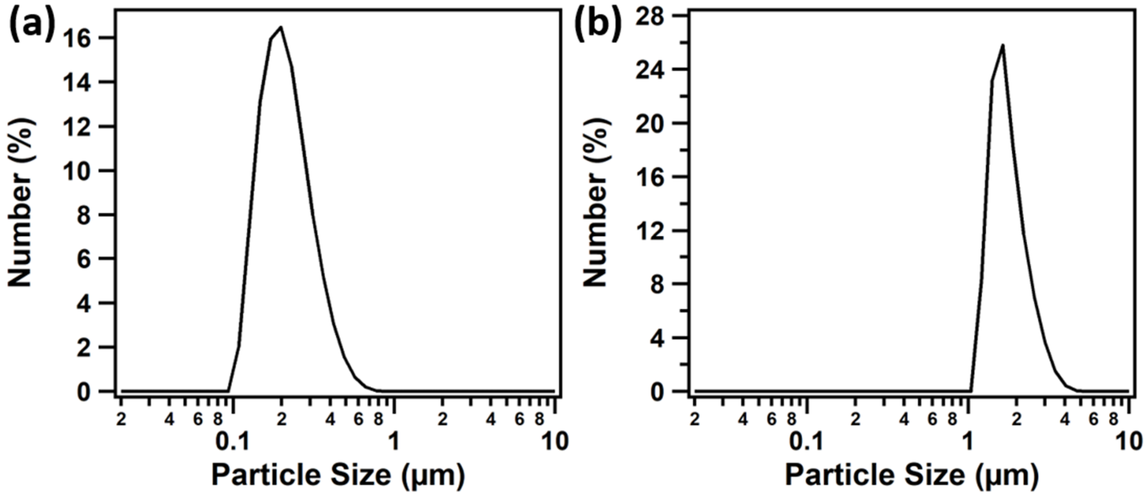

Supplementary Figure 2. Particle size distribution as deduced from dynamic light scattering of halloysite nanotubes dispersed in Milli-Q water (a) before and (b) after adding dopamine.

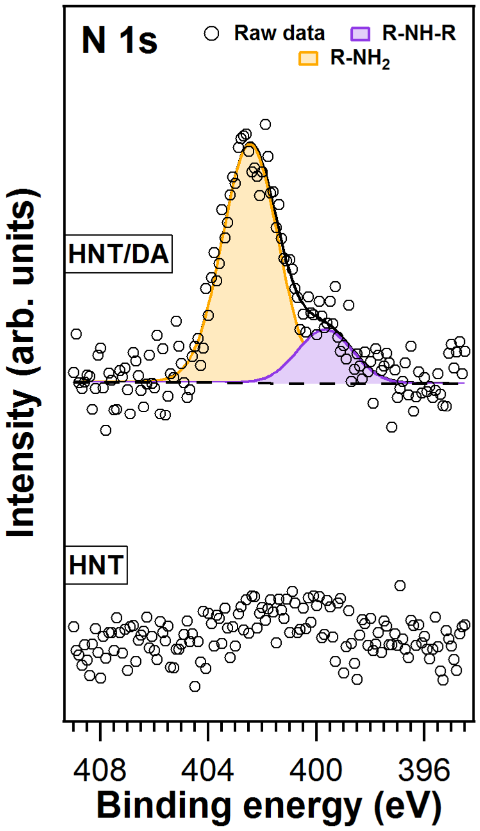

Supplementary Figure 3. XPS spectra of N1*s* core level region before and after dopamine adsorption on HNTs.

To investigate the effects of HNTs on the intermediates' adsorption, UV-vis absorption spectroscopy was performed in two sets of experiments, namely PDA (where the solution contains dopamine and TRIS) and HNT-PDA (where HNTs were added in the reaction medium). Supernatants of reaction mixtures were filtered using 0.2 µm PTFE membranes before measurement in order to prevent scattering caused by insoluble particles such as HNTs or PDA particles. Supplementary Fig. 4 shows the time-dependent UV-vis spectrophotometric changes in the supernatants of the solutions in which PDA and HNT-PDA were formed. The initial dopamine solution shows an absorption band peaked at 281 nm (see dashed line). Upon adjusting the pH of the solution to 8.5 by adding TRIS, a new absorption band at 302 nm was observed in both experiments. As the reaction continues, the intensity of this absorption band decreases, and this reduction is accompanied by a continuous drop absorption at 281 nm. In addition, the general absorption intensity in the range of 310 to 350 nm increased during the polymerization in the case of PDA, while in the case of HNT-PDA no significant absorption was observed in this range (Supplementary Fig. 4a-c). More interestingly, in the visible range PDA and HNT-PDA systems displayed markedly different features (Supplementary Fig. 4b-d). In the case of PDA, a broad absorption peak at 475 nm appears in the initial stage of the reaction but is gradually replaced by a peak at 410 nm as the reaction proceeds. A similar observation was reported also by Herlinger *et al.*[5]. However, in the case of HNT-PDA no remarkable change in the visible range was observed. By attributing the absorption band at 281 nm to dopamine[6], the ones at 302 and 475 nm to dopaminechrome[7] and the one at 420 nm to polydopamine

aggregates[8,9], it can be concluded that in both experiments sets, the rapid conversion of dopamine to dopaminechrome occurs in solution, since the dopaminechrome feature can be seen in the early stages of the reaction. In the case of PDA, the intensity decrease of the dopaminechrome absorption accompanied by gradually increasing wide absorption bands in the UV-Vis range (310-500 nm), centred at 420 nm, indicates that the dopaminechrome moieties gradually transform into weakly soluble oligomers (*e.g.* DHI moieties) and eventually into black insoluble PDA aggregates as the reaction proceeds. However, when HNTs are present in the reaction medium, soluble oligomers are adsorbed on the HNT surface in the early stages of the reaction, leading to a slower formation rate of less soluble oligomers with a wide absorption band in the visible range. These findings support the idea that the presence of HNTs in the polydopamine reaction slows the formation of floating oligomers due to the high rate of adsorption, and confirms the data obtained from the DLS analysis. To reveal the effects of HNTs on the adsorption of the intermediates, the ratio between the absorption intensities at 302 nm and 281 nm was considered as an indication of the relative concentration of dopamine to dopaminechrome. As shown in Supplementary Fig. 5, this ratio remains almost constant at an equilibrated value in the case of PDA, implying that dopamine and dopaminechrome are consumed at relatively similar rates during polymerization. However, when HNTs are present in the reaction medium this ratio continuously decreases as the reaction proceeds, which is an indication of the adsorption of dopaminechrome on the surface of nanotubes. This can be also verified by considering the colour of the supernatants as shown in the inset of Supplementary Fig. 5. Considering that dopaminechrome containing oligomers are responsible for the orange colour of the solution[10,11], the lighter orange colour of supernatant in the case of HNT-PDA compared with PDA confirms the lower presence of this structure in the former.

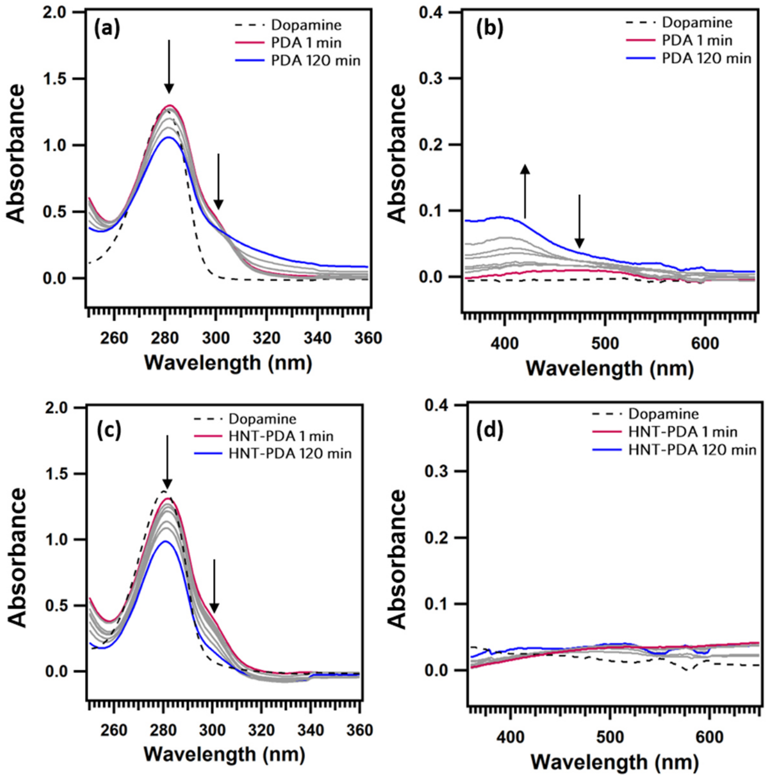

Supplementary Figure 4. UV-vis absorption spectra of the supernatant of dopamine polymerization without the presence of HNTs(a and b) and with HNTs (c and d) in the reaction medium.

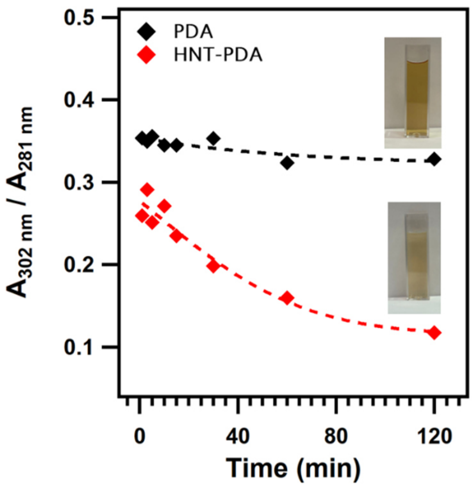

Supplementary Figure 5. Ratio between the UV-vis absorption signals of dopaminechrome and dopamine in the solution (inset photographs showing the supernatants of the reaction mixtures after 120 min of reaction)

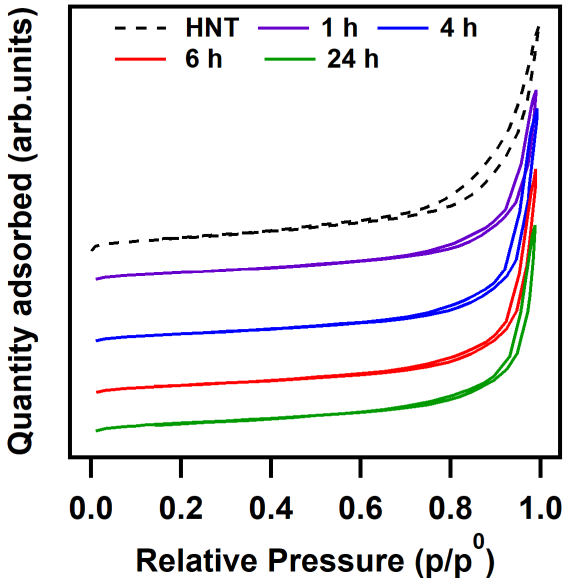

Supplementary Figure 6. N_2 adsorption/desorption isotherms for HNT-PDA after different polymerization times.

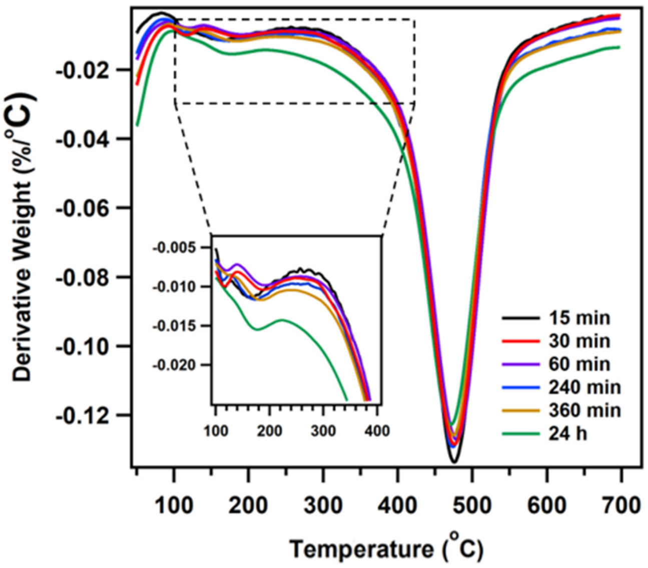

Supplementary Figure 7. Derivative thermogravimetric graphs for HNT-PDA after different polymerization times.

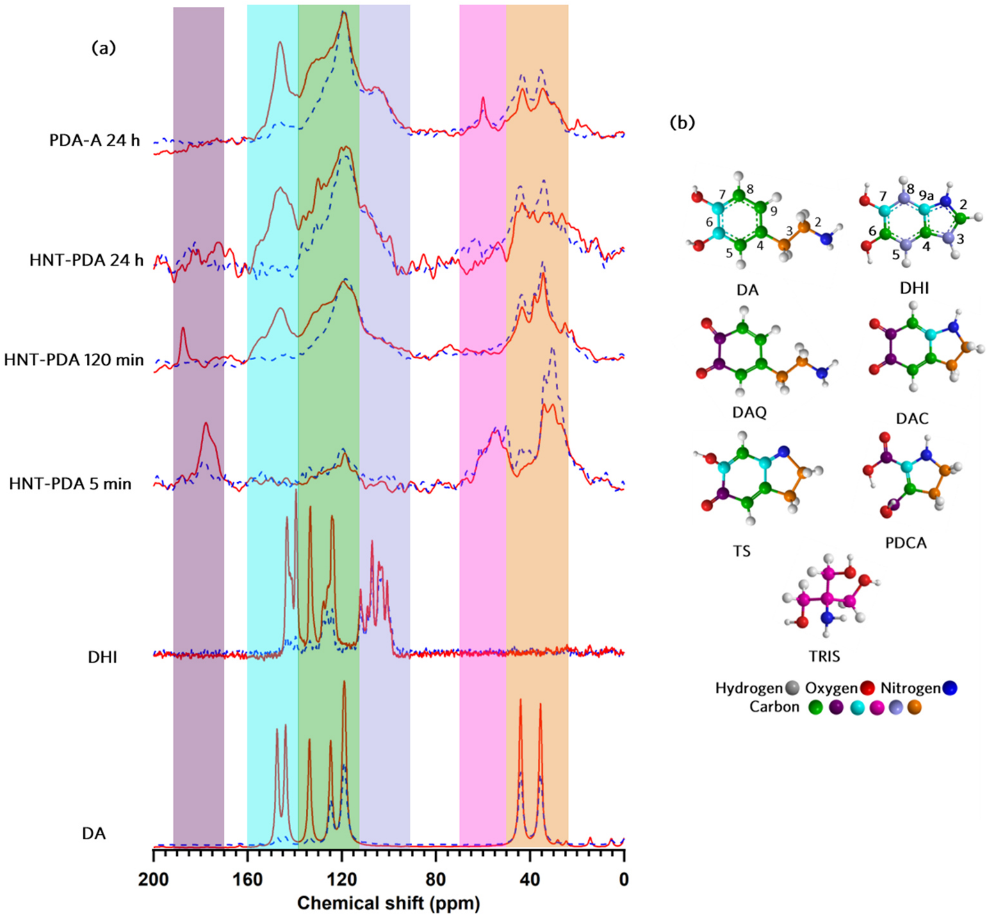

Supplementary Figure 8. (a) Carbon-13 CP MAS solid-state NMR spectra for dopamine, DHI, polydopamine deposited on the HNTs surface (HNT-PDA) after different reaction times, and polydopamine obtained in the absence of HNTs (PDA-A); (b) chemical structures of the

expected building blocks of polydopamine, in which specific carbon types with characteristic ^{13}C chemical shifts are marked with different colours (see legend at bottom). The same colour codes are marked in the background layer of panel (a). The ssNMR data were measured at 18 kHz MAS, and at 600 MHz (for ^{1}H), using ramped CP ssNMR with short (80 µs, dashed blue lines) and long (2 ms, red) CP contact times.

A more in-depth analysis was performed, by measuring the ^{13}C ssNMR spectra of the HNT-PDA at different reaction times, and comparig these to the spectra of dopamine and DHI. Supplementary Fig. 8b shows the structures of the most considered building blocks in the literature; the colour allocation represents the relative chemical shift in the ssNMR analysis. The carbon assignments for dopamine and DHI molecules were previously reported by Ni et al.[12], D'Ischia et al.[13,], and Murphy et al.[14], respectively. Supplementary Fig. 8a displays the ^{13}C CP ss-NMR spectra of dopamine (DA) (as the starting molecule), DHI and PDA-A (as the control sample), and HNTs after different polydopamine reaction times, i.e. 5 min, 2 and 24 h. All spectra were acquired by ramped ^{1}H-^{13}C CP with short (80 µs, red lines) and long (2 ms, blue) contact times. It should be noted that the signal to noise ratio of the ssNMR spectra on HNTs samples is limited by the low weight percentage of the organic layer deposited on the nanotubes (reaches only 5% after 24 h of the reaction, as demonstrated by the TGA analysis). In the ssNMR spectra of the initial dopamine monomer (DA) two well resolved resonances in the range of 30-50 ppm are attributed to the protonated aliphatic carbons at positions 2 and 3 which are identified by orange colour. Two quaternary carbons bonded to oxygen in catechol form at positions 6 and 7 and resonate in the range of 140-160 ppm (coloured in light blue) while the aromatic carbons at positions 4, 5,8 and 9 resonate in the range of 110-140 ppm (coloured in green). The lower intensity of resonance at around 130 ppm in the low contact time spectrum indicates that this signal belongs to the quaternary carbon at position 4 of the dopamine structure[13,14]. In the ssNMR spectra of DHI, three protonated aromatic carbons at positions 3, 5 and 8 show a broad resonance in the range of 90-110 ppm which is coloured in lavender. The other protonated carbon at position 2 resonates at ~122 ppm. The resonance of this carbon is detected in the long contact time spectrum of DHI while in the short contact time it is less pronounced. This might be due to the dimerization of the DHI molecule on 2 position. Indeed, it has been showed that the C-C connection between two DHI molecules on 2 position is energetically favorable which alters the carbon state from protonated to quaternary[15,16]. Considering the quaternary carbons, two carbons adjacent to electronegative atoms (oxygen and nitrogen) at positions 7 and 9a (coloured in light blue) show downfield resonances in the range of 140-160 ppm. Two other quaternary carbons at positions 4 and 6 resonate in the range of 110-140 ppm (coloured in green)[13,14]. After 5 min of dopamine polymerization in the presence of HNTs, the spectrum changes dramatically and was not similar to dopamine and DHI. Two new resonances at~60 ppm belonging to the protonated carbon of the TRIS structure (coloured in pink) and at ~175 ppm ascribed to the quaternary carbons in the carbonyl form (coloured in purple) are clearly observed. These observations, along with the absence of any resonance in the range of 140-160 ppm, indicate that the

primary adsorbed structures mainly contain dopaminequinone (DAQ) and TRIS structures. As the reaction continues, a new resonance was detected in the range of 140-160 ppm, which is attributed to the bridgehead carbon atom bonded to nitrogen in DAC, TS and PDCA structures and to catechol moieties in DA and TS structures. The appearance of this resonance together with the presence of the carbonyl carbon resonance in the range of 170-190 ppm (coloured in purple) indicates that the initially formed DAQ structure undergoes a gradual cyclization reaction which leads to the formation of the DAC structure. Although DHI structure also shows resonance in the range of 140-160 ppm, the low visibility of its other characteristic resonances in the range 90-110 ppm implies that the DHI structure does not play a significant role in the initial stages of polydopamine film formation. After 24h of the polydopamine deposition the intensity of the resonance in the range of the carbonyl moieties decreases while the intensity of the resonances in the range of 90-110 ppm increases. This may point to the further conversion of DAC molecules to DHI through a dehydrogenation reaction in the indoline ring. However, as this is observed in the late stage of reaction, we can conclude that the conversion of DAC to DHI is a relatively slow reaction and DHI does not contribute in the early stages of polydopamine film formation. The same observation has been reported in some other previous studies[17,18]. Moreover, it can be seen that the intensity of the resonance in the range of 90-110 ppm belonging to DHI is still less pronounced compared to that of PDA aggregates that were obtained in the absence of HNTs. Indeed, when HNTs are not present in the reaction medium DHI has a great role in the PDA aggregation process because of its low solubility in the reaction medium. However, in the case of HNTs, the negatively charged HNTs surface plays an important role in capturing the soluble intermediates from the solution. In addition, DHI has an extremely weak basic character [19] and does not bring positive charge in the reaction medium, which results in the less favourable adsorption of this molecule on the negatively charged nanotubes. For this reason, it is reasonable to infer that DHI should not have a high contribution in the structure of the PDA formed on HNTs in the time window of our XPS study.

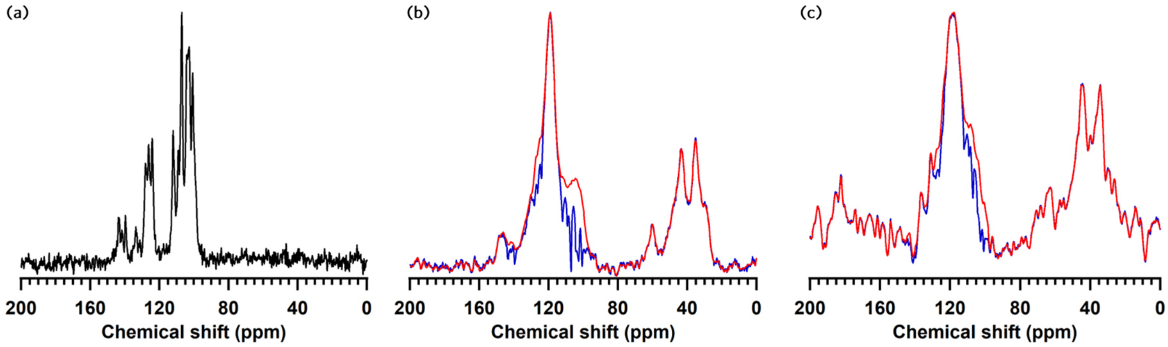

Supplementary Figure 9. Carbon-13 cross-polarization magic angle spinning nuclear magnetic resonance spectra recorded at 18 kHz MAS frequency by using standard CP-MAS sequence with a contact time of 80 µs, for (a) DHI, (b) polydopamine obtained in the absence of HNTs after 24 h of reaction (PDA-A) and (c) polydopamine deposited on HNTs after 24 h of reaction (HNT-PDA). The red line represents the original spectra and the blue line represents the spectra obtained after subtracting DHI spectrum from the representative spectra.

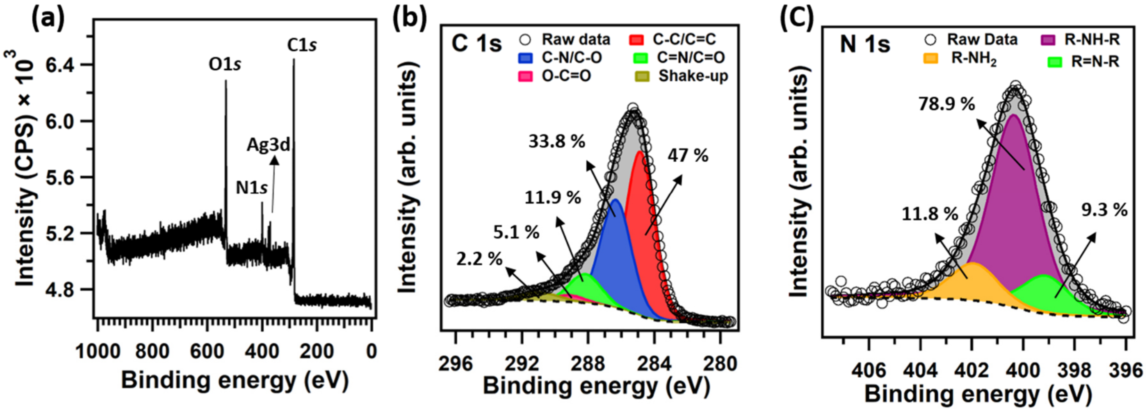

Supplementary Figure 10. X-ray photoelectron spectroscopy: (a) wide scan spectrum, (b) C1*s* and (c) N1s core-level regions of PDA-A.

To have a reference, we first investigated by XPS the surface chemical composition of the PDA-A sample, isolated from an aqueous alkaline dopamine solution after 24 h. The results are shown in Supplementary Fig. 10; carbon, nitrogen and oxygen in respectively atomic percentages of 70.5, 22.1 and 7.4 at.% were identified as the main constituents of PDA-A sample. The doublet signal located at binding energies (BE) of 373.8 and 367.8 eV is respectively attributed to the Ag3$d_{3/2}$ and Ag3$d_{5/2}$ core levels from the silver substrate, which was used as a support for mounting the samples (Supplementary Fig. 10a). The C1*s* spectrum (Supplementary Fig. 10b) exhibits an asymmetrical shape, which requires five components to obtain a good fit. The peak at a BE of 284.8 eV (marked in red) can be assigned to C-C/C=C species, while the other components located at 286.1, 287.5, 289.2 eV can be ascribed to C-O/C-N (blue), C=O/C=N (green) and O=C-O (pink) bonds, respectively[20,21]. The peak at about 291.3 eV is assigned to the shake-up component[22]. The presence of the carbonyl functional group indicates that a portion of the catechol groups in dopamine units converted to quinone groups upon auto-oxidation, as already reported in previous studies[23]. Moreover, the presence of the O=C-O component in the C1*s* core level region confirms the oxidative breakage of the quinone rings by the H_2O_2 formed *in situ*, resulting in the formation of pyrroline dicarboxylic acid moieties (PDCA)[24]. The N1*s* spectrum (Supplementary Fig. 10c) evidences the presence of three chemical environments for nitrogen atoms. The predominant peak, located at a BE of 400.3 eV is attributed to the secondary amine moieties (R-NH-R, marked in purple), while the two less intense components located at BEs of 402.0 and 399.0 eV are assigned to primary amine (R-NH_2, orange) and imine (R=N-R, green) functional groups, respectively[22]. Considering the most proposed building blocks for PDA, primary amine corresponds to both DA and DAQ, secondary amine is associated with DAC, DHI and PDCA and imine is associated with the tautomeric species, TS (see Fig. 4 in the main text). The predominance of the secondary amine species points out the crucial role of the conversion of dopamine molecules into the reaction intermediates and tautomeric species by oxidation and cyclization, in agreement with the previously reported mechanism for the PDA formation[23,25].

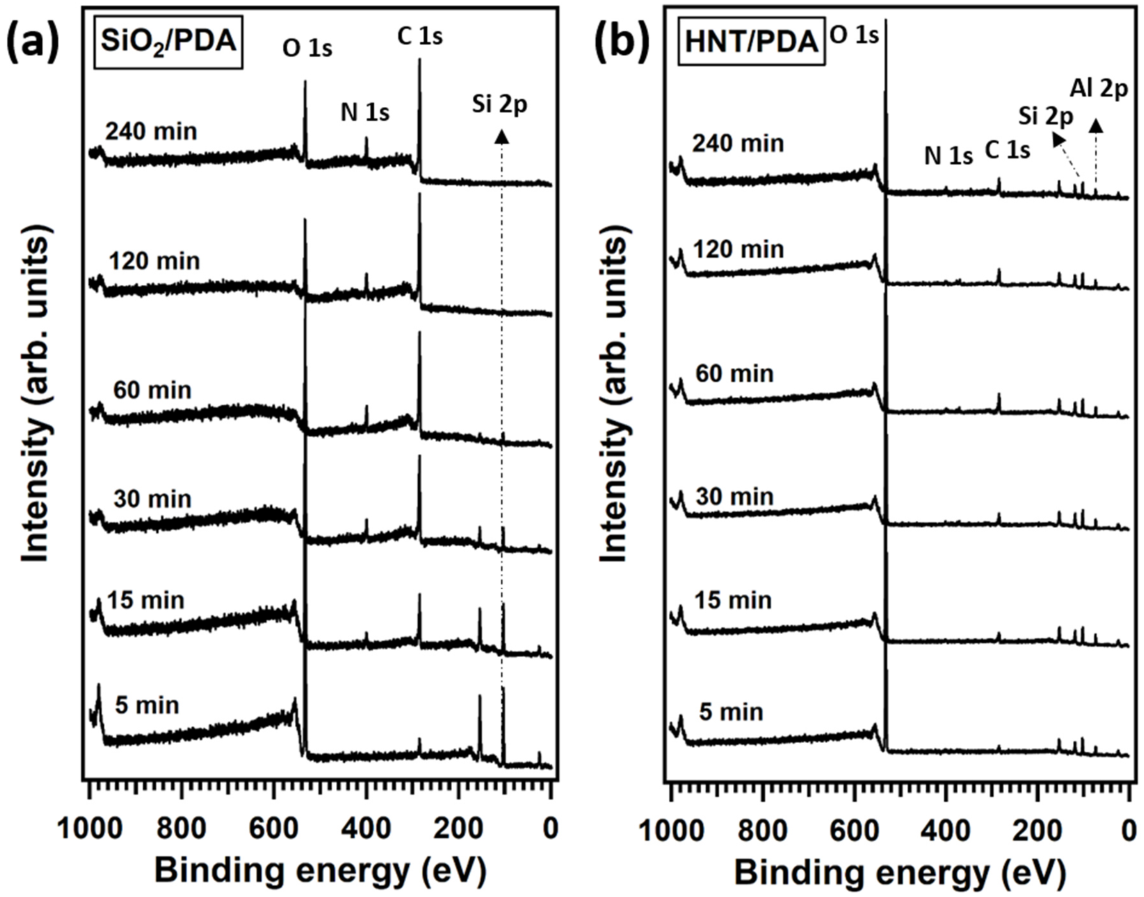

Supplementary Figure 11. X-ray photoelectron spectroscopy: wide scan surveys for a PDA layer deposited on (a) SiO_2 and (b) HNTs after different dopamine polymerization times.

Supplementary Fig. 12 shows the C1*s* and N1*s* core level spectra of HNTs after 5 and 15 min of dopamine polymerization in the presence of a carbonate buffer instead of a TRIS buffer. Similarly to what was observed in the TRIS case, in both samples the C1*s* spectra can be fitted with five components; C-C/C=C (marked in red in Supplementary Fig. 12a-b, observed at a binding energy (B.E.) of 284.8 eV), C-O/C-N (blue, B.E.=286.1 eV), C=O/C=N (green, B.E.=287.5 eV), O=C-O (pink, B.E.=289.2 eV) bonds and a shake-up component at a B.E. of 291.3 eV. However, in both samples, the relative ratio between the intensities due to the C-N/C-O and C-C/C=C components is less than 1, in contrast to the samples prepared using TRIS. In addition, the same chemical species whose spectroscopic fingerprint was observed in the N1*s* spectrum of the samples which were prepared in the presence of TRIS, can be identified in the N1*s* spectra of the samples obtained in the presence of carbonate, namely $R\text{-}NH_3^+$ (marked in orange in Supplementary Fig. 12c-d, B.E.=402.5 eV) and R-NH-R (purple, B.E.=400.1 eV). It can be seen that the signal due to primary amine is not the main feature of the N1*s* core level line, contrary to what observed in the samples prepared with TRIS. Overall, these observations support the incorporation of the TRIS molecule into the initial oligomers, as we already concluded from ss-NMR studies

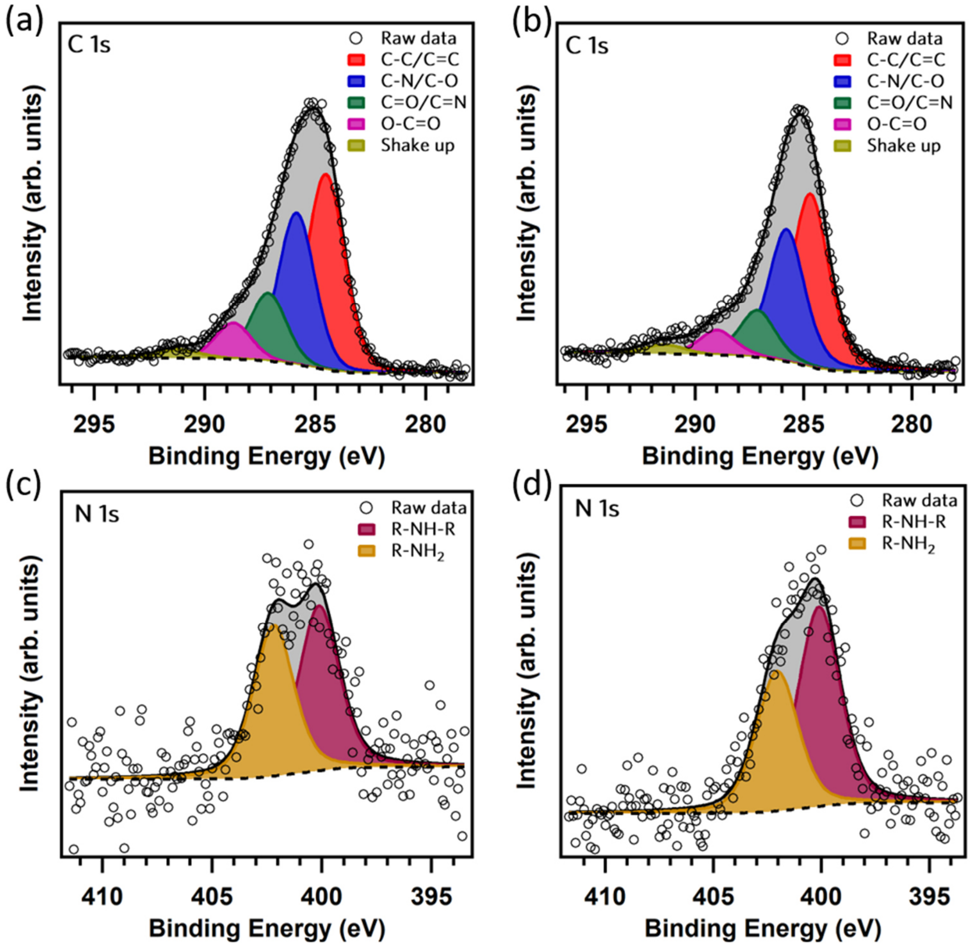

Supplementary Figure 12. C1*s* core level spectra for (a) HNT-PDA 5 and (b) HNT-PDA 15 samples obtained using a carbonate buffer. N1*s* core levels spectra for (c) HNT-PDA 5 and (d) HNT-PDA 15 samples obtained using a carbonate buffer.

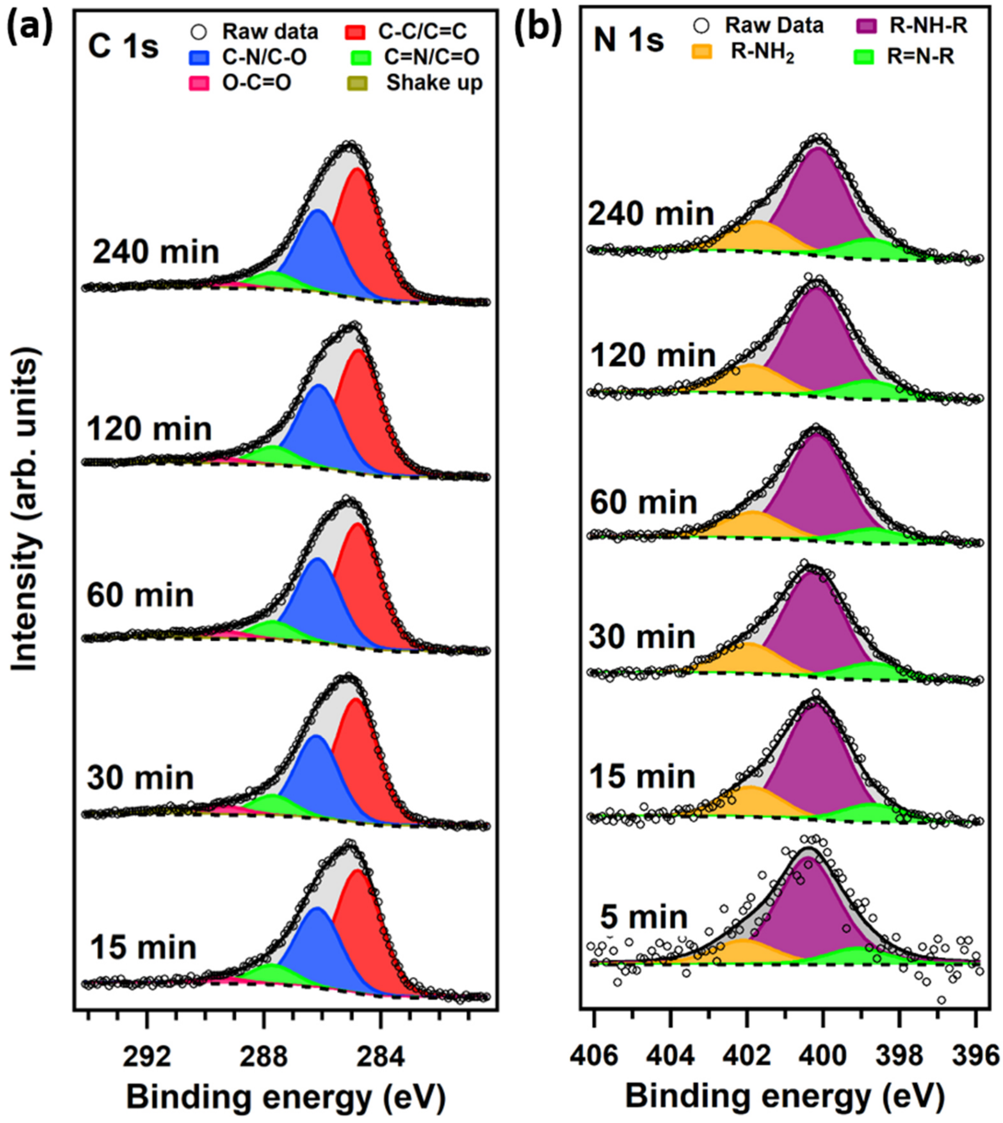

Supplementary Figure 13. XPS High-resolution spectra of (a) C1*s* and (b) N1*s* core level regions of the PDA layer deposited on a SiO_2 substrate after different deposition times.

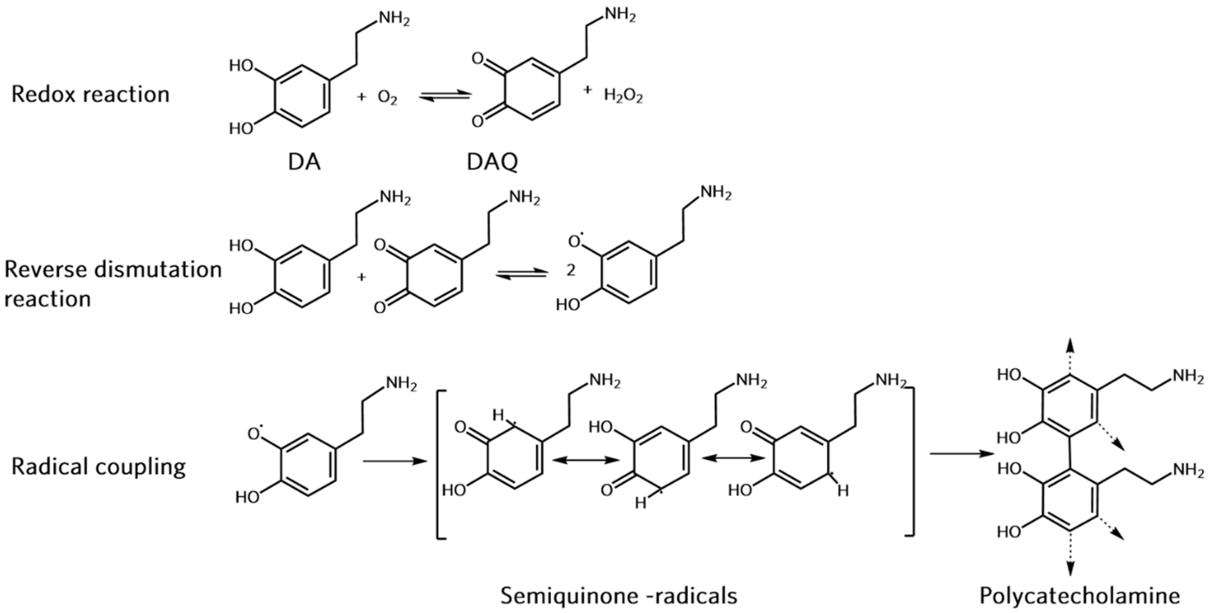

Supplementary Figure 14. The reaction pathway for cross-linking of quinone and dopamine units resulting in formation of polycatecholamines[26].

To further study the intermediates structures adsorbed on the nanotubes Uv-Vis diffusive reflection spectroscopy on the nanotubes in the solid state was performed. The reflectance data were transformed to the absorbance using Kubelka-Munk function and the data are displayed in Supplementary Fig. 15a-c. The absorption spectrum of the pristine nanotubes was acquired and is shown as control sample. As it can be seen in the Supplementary Fig. 15a, the absorption spectrum of HNTs significantly changes after modification with PDA. Samples with longer exposure time to the reaction solution show a higher general absorption in the visible range, which is consistent with a gradual change in samples colour from white for pristine HNTs to dark grey for HNT-PDA 4h (Supplementary Fig. 15d). In all samples, the absorption band of DAQ (at ~ 350 nm)[27,28] was detected on the nanotubes after modification with polydopamine. It is important to note that although DAQ structure was not detected in UV-Vis spectra of the reaction's supernatant, it is observed in the UV-Vis spectra acquired on the nanotubes. This can be attributed to the unstable nature of DAQ structure in the alkaline solutions, which does not allow us to detect this structure by studying the Uv-Vis spectra of the supernatants. In addition, the absorption band of DAC (at 475 nm) is detected in all samples. The other absorption band of this structure at 302 nm cannot be resolved due to its overlap with the absorption line of nanotubes. Supplementary Fig. 15b-c show the relative absorption intensities for the DAQ and DAC structures adsorbed on the nanotubes after various reaction times. Similar to what was suggested in the manuscript based on the XPS analysis and further confirmed by ss-NMR analysis, DAQ adsorption is high on the initial samples and then gradually decrease with increasing the polymerization time, while the DAC adsorption continuously increases as the reaction proceeds. Overall, these data provide further supports for the polydopamine formation mechanism that we propose.

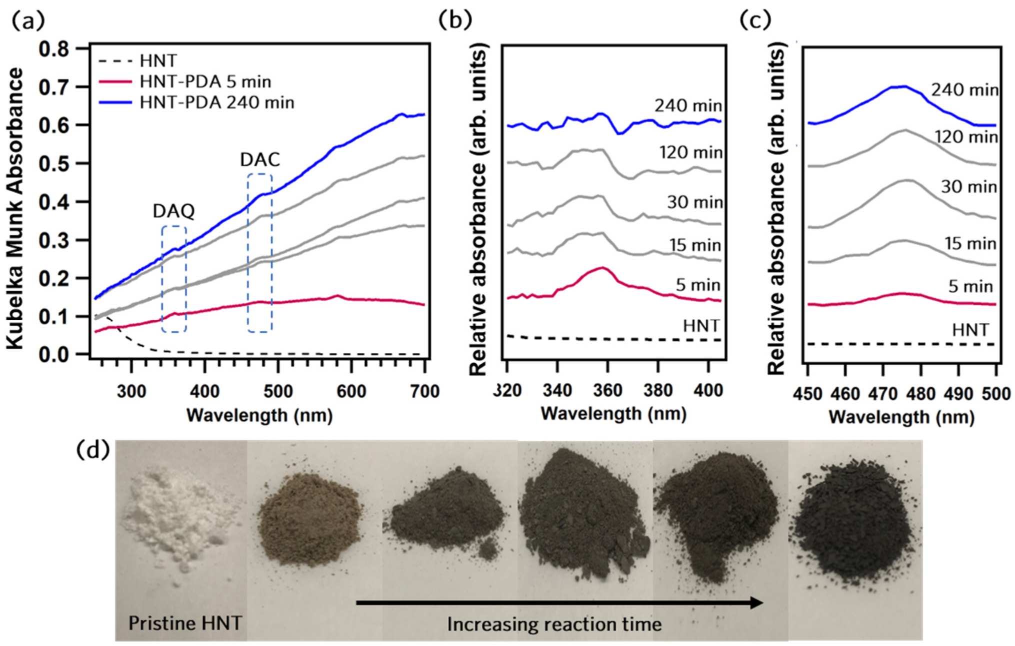

Supplementary Figure 15. (a) Reflective UV-vis spectra of HNT-PDA after varied polymerization times, relative absorbance of (b) dopaminequinone and (c) dopaminechrome adsorbed on the nanotubes, (d) colour change of HNTs after varied polymerization times.

Supplementary Tables

Supplementary Table 1. Atomic percentages for the scanned area from EDS analysis (±1.0%)

Sample	Si	Al	O	C
HNT-PDA 1h	15.1	15.0	66.9	3.0
HNT-PDA 4h	13.6	13.6	65.8	7.0
HNT-PDA 24h	13.1	13.0	54.4	19.5

Supplementary Table 2. Chemical composition (in at.%) of SiO_2-PDA and HNT-PDA after different polymerization times and corresponding N/C ratio.

Polymerization time (min)	Chemical composition (in at.%)										
	SiO_2-PDA					HNT-PDA					
	C	N	O	Si	N/C	C	N	O	Al	Si	N/C
5	7.5	0.7	63.1	28.7	0.09	7.3	0.9	64.5	13	14.3	0.12
15	27.9	3.1	47.7	21.3	0.11	9.5	1	63.3	12	14.2	0.10
30	55.0	5.9	28.4	10.7	0.11	11	1.2	62.1	11.7	14	0.11
60	66.7	8.0	21.0	4.3	0.12	15.8	1.5	58	11.4	13.3	0.09
120	71.6	7.9	19.5	1.0	0.11	15.3	1.6	60	10.5	12.6	0.10
240	73.5	8.1	18.4	0	0.11	17.4	2	60.1	9.5	11	0.11

Supplementary Table 3. Relative contribution (in %) of the different functional groups to the total spectral intensities of the C1*s* and N1*s* photoemission lines of the PDA layer deposited on the SiO_2 substrate at different deposition times.

Deposition time (min)	C1*s*					N1*s*		
	C-C,C=C	C-N,C-O	C=O,C=N	O=C-C	Shake-up	$R\text{-}NH_3^+$	R-NH-R	R=N-R
5	50.2	37.4	10.9	1.5	0	16.6	72.1	11.3
15	54.0	35.0	8.5	2.5	0	18.3	70.7	11.0
30	51.5	34.4	8.4	3.6	2.1	19.7	69.1	11.2
60	51.8	35.2	7.7	3.2	2.1	17.8	72.4	9.8
120	53.3	35.5	7.5	2.3	1.4	18.1	70.0	11.9
240	55.0	34.8	7.0	2.1	1.1	19.1	68.1	12.8

Supplementary Table 4. Numbers of chemical species in each building block of polydopamine

Molecule	Chemical Species						
	C-C/C=C	C-O/C-N	C=O/C=N	O-C=O	R-NH-R	R-NH_3^+	R=N-R
Dopamine (DA)	5	3	0	0	0	1	0
Dopamine quinone (DAQ)	5	1	2	0	0	1	0
Dopaminechrome (DAC)	4	2	2	0	1	0	0
Tris	0	4	0	0	0	1	0
Pyrroline dicarboxylic acid (PDCA)	2	2	0	2	1	0	0
Tautomeric structure (TS)	4	2	2	0	0	0	1

$$\frac{5\,DA + 5\,DAQ + 4\,DAC + 2\,PDCA + 4\,TS}{3\,DA + 1\,DAQ + 2\,DAC + 4\,Tris + 2\,PDCA + 2\,TS} = \frac{C{-}C, C{=}C}{C{-}O, C{-}N} \tag{1}$$

$$\frac{3\,DA + 1\,DAQ + 2\,DAC + 4\,Tris + 2\,PDCA + 2\,TS}{2\,DAQ + 2\,DAC + 2\,TS} = \frac{C{-}O, C{-}N}{C{=}O, C{=}N} \tag{2}$$

$$\frac{3\,DA + 1\,DAQ + 2\,DAC + 4\,Tris + 2\,PDCA + 2\,TS}{2\,PDCA} = \frac{C{-}O, C{-}N}{O{-}C{=}O} \tag{3}$$

$$\frac{DAC + PDCA}{DA + DAQ + Tris} = \frac{R{-}NH{-}R}{R{-}NH_3^+} \tag{4}$$

$$\frac{TS}{DA + DAQ + Tris} = \frac{R{=}N{-}R}{R{-}NH_3^+} \tag{5}$$

$$DA + DAC + DAQ + Tris + PDCA + TS = 1 \tag{6}$$

Empirical model developed based on the X-ray photoelectron spectroscopy data

To evaluate the percentage of each building block during the dopamine polymerization, a numerical method was used. By taking into account the number of the chemical species present in each building block structure (see Fig. 4 in the main text and Supplementary Table 4) the left side of the equations 1 to 6 were developed, while for the right side the ratios were obtained from the relative intensities of the various chemical groups in the high-resolution spectra of the C1*s* and N1*s* core level regions; the partial presence of each building block is shown by the representative abbreviations. Specifically, considering equation (1), the right hand side corresponds to the ratio between the spectral intensities of the C-C/C=C species (marked in red in Fig. 5 of the main text) and that of the C-O/C-N (blue in Fig. 5 main text). The six equations system was solved simultaneously, using a linear equations solver for six

variables for each of the polymerization times for which we had collected XPS data and the results are summarized in Supplementary Table 5.

Supplementary Table 5. Evolution of the building blocks in HNT-PDA (in %) as a function of polymerization time.

Building blocks	Polymerization time (min)					
	5	15	30	60	120	240
Dopamine (DA)	0	3.0	18.6	18.5	13.0	7.4
Dopamine quinone (DAQ)	30.6	17.5	12.9	2.1	4.3	0
Dopaminechrome (DAC)	11.6	23.0	18.1	26.4	32.7	44.0
TRIS	48.2	44.0	31.7	27.2	26.8	24.0
Pyrroline dicarboxylic acid (PDCA)	9.6	12.5	12.2	19.0	16.1	15.0
Tautomeric structure (TS)	0	0	6.5	6.8	7.1	9.6

Supplementary Table 6. Comparison of the ratios between chemical species as deduced from the XPS data and as predicted by the model.

HNT-PDA samples	Chemical species ratio			
	C-C, C=C/C=O, C=N		C-C,C=C/O-C=O	
	Model	Experimental Data	Model	Experimental data
5 min	2.6	2.0	11.4	11.4
15 min	2.7	2.4	8.8	8
30 min	3.7	3.8	11.5	11.3
60 min	3.9	3.8	7.2	7.1
120 min	3.1	3.1	8.6	8.5
240 min	2.6	2.7	9.4	9.1

Supplementary Table 6 shows a comparison of the ratios between different chemical species, which were not taken into account in equations 1 to 6, calculated based on experimental data versus those predicted by the model. One clearly sees that the experimental data match well with the model, and hence validate it.

Supplementary References

1. Lim, S. *et al*. Modification of halloysite nanotubes for enhancement of gas-adsorption capacity. *Clays Clay Miner.* **68,** 189-196 (2020).

2. Wan, X. *et al*. Core@double-shell structured magnetic halloysite nanotube nano-hybrid as efficient recyclable adsorbent for methylene blue removal. *Chem. Eng. J.* **330,** 491-504 (2017).

3. Kohyama, N. *et al*. Observation of the hydrated form of tubular halloysite by an electron microscope equipped with an environmental cell. *Clays Clay Miner.* **26,** 25-40 (1978).

4. Hemmatpour, H. *et al*. Synthesis of pH-sensitive poly (N, N-dimethylaminoethyl methacrylate)-grafted halloysite nanotubes for adsorption and controlled release of DPH and DS drugs. *Polymer* **65,** 143-153 (2015).

5. Herlinger, E. *et al*. Spontaneous autoxidation of dopamine. *J. Chem. Soc., Perkin trans.* **2**, 259-263 (1995).

6. Cui, R. *et al*. Determination of $Fe3^{+}$ upon special "upconversion luminescence" of dopamine. *ACS Omega* **4(6)**, 9918-9924 (2019).

7. Di, J. *et al*. Effect of aluminum (III) on the conversion of dopachrome in the melanin synthesis pathway. *SPECTROCHIM. ACTA A*. **59(8)**, 1689-1696 (2003).

8. Cihanoğlu, A. *et al*. Ultrasound-assisted dopamine polymerization: rapid and oxidizing agent-free polydopamine coatings on membrane surfaces. *ChemComm* **57(100)**, 13740-13743 (2021).

9. Qu, X. *et al*. Selective extraction of bioactive glycoprotein in neutral environment through Concanavalin A mediated template immobilization and dopamine surface imprinting. RSC *Adv.* **6(89)**, 86455-86463 (2016).

10. Solano, F. *et al*. Neurotoxicity due to o-quinones: neuromelanin formation and possible mechanisms for o-quinone detoxification. *Neurotox. Res.* **1(3)**, 153-169 (1999).

11. Noh, M. Y. *et al*. Cuticle formation and pigmentation in beetles. *Curr. Opin. Insect. Sci*, **17**, 1-9 (2016).

12. Ni, Q. Z. *et al*. Chemoenzymatic elaboration of the Raper–Mason pathway unravels the structural diversity within eumelanin pigments. *Chem. Sci. J.* **11(30)**, 7836-7841 (2020).

13. D'Ischia, M. *et al*. 5, 6-dihydroxyindoles and indole-5, 6-diones. *Adv. Heterocycl. Chem.* **89**, 1-63 (2005).

14. Murphy, B. P. *et al.* Synthesis and physical properties of 5, 6-dihydroxyindole. *J. Org. Chem* , **50(15)**, 2790-2791 (1985).

15. Chen, C. T. *et al*. Polydopamine and eumelanin models in various oxidation states. *Phys. Chem. Chem.* **20(44)**, 28135-28143 (2018).

16. Chen, C. T. *et al*. Polydopamine and eumelanin molecular structures investigated with *ab initio* calculations. *Chem. Sci*. **8(2)** 1631-1641 (2017).

17. Lyu, Q. *et al*. *In situ* insights into the nanoscale deposition of 5, 6-dihydroxyindole-based coatings and the implications on the underwater adhesion mechanism of polydopamine coatings. *RSC Adv*. **8(49)**, 27695-27702 (2018).

18. Lyu, Q. *et al*. Direct evidence for the critical role of 5, 6-dihydroxyindole in polydopamine deposition and aggregation. *Langmuir* **35(15)**, 5191-5201 (2019).

19. Wishart, D.S. *et al*. HMDB 4.0 — The Human Metabolome Database for 2018. *Nucleic Acids Res*. **46**, D608-17 (2018).

20. Ding, Y. *et al*. Insights into the aggregation/deposition and structure of a polydopamine film. *Langmuir* **30(41),** 12258-12269 (2014).

21. Feng, J. *et al*. Characterizations of the formation of polydopamine-coated halloysite nanotubes in various pH environments. *Langmuir* **32(40),** 10377-10386 (2016).

22. Zangmeister, R. A. *et al*. Characterization of polydopamine thin films deposited at short times by autoxidation of dopamine. *Langmuir* **29(27),** 8619-8628 (2013).

23. Liebscher, J. Chemistry of polydopamine–scope, variation, and limitation. *Eur. J. Org. Chem*. **2019,** 4976-4994 (2019).

24. Cheng, W. *et al*. Versatile polydopamine platforms: synthesis and promising applications for surface modification and advanced nanomedicine. ACS Nano **13(8),** 8537-8565 (2019).

25. Lyu, Q. *et al*. Unravelling the polydopamine mystery: is the end in sight? *Polym. Chem.* **10(42),** 5771-5777 (2019)

26. Burzio, L. A. *et al*. Cross-linking in adhesive quinoproteins: studies with model decapeptides. *Biochem.* **39(36)**, 11147-11 (2000).

27. Wei, Q. *et al*. Oxidant-induced dopamine polymerization for multifunctional coatings. *Polym. Chem.* **1(9)**, 1430-1433 (2010).

28. Chen, T. P. *et al*. Self-polymerization of dopamine in acidic environments without oxygen. *Langmuir* **33(23),** 5863-5871 (2017).
